# Transgenic mice overexpressing the LH receptor in the female reproductive system spontaneously develop endometrial tumour masses

**DOI:** 10.1038/s41598-021-87492-5

**Published:** 2021-04-23

**Authors:** Tiziano Lottini, Jessica Iorio, Elena Lastraioli, Laura Carraresi, Claudia Duranti, Cesare Sala, Miriam Armenio, Ivo Noci, Serena Pillozzi, Annarosa Arcangeli

**Affiliations:** 1 Department of Experimental and Clinical Medicine, Section of Internal Medicine, University of Florence, Viale G.B. Morgagni, 50, 50134 Florence, Italy; 2 DI.V.A.L. Toscana srl, Sesto Fiorentino, Florence, Italy; 3 Department of Biochemical, Experimental and Clinical Science, University of Florence, Florence, Italy; 4 CSDC-Center for the Study of Complex Dynamics, 50019 Sesto Fiorentino, Florence, Italy

**Keywords:** Biological techniques, Cancer, Cell biology, Biomarkers, Diseases, Oncology, Pathogenesis, Risk factors

## Abstract

The receptor for the luteinizing hormone (LH-R) is aberrantly over expressed in cancers of the reproductive system. To uncover whether LH-R over expression has a causative role in cancer, we generated a transgenic (TG) mouse which overexpresses the human LH-R (hLH-R) in the female reproductive tract, under the control of the oviduct-specific glycoprotein (OGP) mouse promoter (*mogp-1*). The transgene was highly expressed in the uterus, ovary and liver, but only in the uterus morphological and molecular alterations (increased proliferation and trans-differentiation in the endometrial layer) were detected. A transcriptomic analysis on the uteri of young TG mice showed an up regulation of genes involved in cell cycle control and a down regulation of genes related to the immune system and the metabolism of xenobiotics. Aged TG females developed tumor masses in the uteri, which resembled an Endometrial Cancer (EC). Microarray and immunohistochemistry data indicated the deregulation of signaling pathways which are known to be altered in human ECs. The analysis of a cohort of 126 human ECs showed that LH-R overexpression is associated with early-stage tumors. Overall, our data led support to conclude that LH-R overexpression may directly contribute to trigger the neoplastic transformation of the endometrium.

## Introduction

The luteinizing hormone receptor (LH-R) is a member of the glycoprotein hormone seven-transmembrane domain receptor family^1^, capable of binding to both LH and hCG, with high affinity. The interaction of LH-R with LH increases the production of cAMP and phospholipase C, hence stimulating ovarian steroidogenesis in granulosa and luteal cells and then triggering ovulation in females^2,3^. In males, the LH/LH-R interaction promotes steroidogenesis in Leydig cells, especially around puberty^4^.

An over-expression of LH-R has been described in different types of cancers of either the male or female reproductive system. Indeed, LH-R is highly expressed on the plasma membrane of hyperplastic and malignant prostate cells and its activation increases the expression of steroidogenic enzymes and hence steroid production^5^. Although the function of LH-R in prostate cancer is not completely clear, the LH/LH-R axis may represent a therapeutic target in this cancer type^6^. LH-R is also overexpressed in Ovarian Cancer (OC) and an increased risk to develop an OC is associated with increased LH serum levels^7^, and increased LH-R levels in OC behave as independent prognostic factors for overall survival^8^. LH-R was also found to be overexpressed in Endometrial Cancer (EC) cells and its activation by LH promotes local cancer spread and metastatic progression, through the activation of protein kinase A (PKA)^9^. LH-R is overexpressed also in primary EC samples^10^ and a high expression level correlates with an early tumor relapse, in a patient with low risk EC^11^. Furthermore, the binding of LH to its receptor increases the metastatic spread of EC cells, in a preclinical menopausal mouse model^12^. Overall, a relationship between high LH-R expression and neoplastic progression in EC is emerging, but the evidence for a causative role of the LH/LH-R axis in the cancerogenic process leading to EC establishment is still lacking.

The role of gene mutations/overexpression in neoplastic transformation can be determined by developing genetically engineered (GEM) mice carrying specific genetic alterations in cancer driving genes^13^. Some GEM models have been generated to study the physiology and the pathophysiology of gonadotropins and their receptors^14,15,16^. From these models it emerged that either an excess or the lack of gonadotropins are involved in tumorigenesis, in germline cells, sex-cord stromal tumors and ovary surface epithelial tumors^16^. The knocking out (KO) of gonadotropin receptors, in particular of LH-R, gives rise to mice with impaired development of the sexual organs^17^.

To elucidate the possible causative role of LH-R overexpression in the neoplastic transformation leading to the establishment of cancers in the female reproductive system, we generated transgenic (TG) mice which express the human LH-R (hLH-R) under the control of the oviduct-specific glycoprotein (OGP) mouse promoter-1 (*mogp-1*), and hence overexpress hLH-R in the female reproductive tract.

## Results

### Generation of LH-R (TG) transgenic mice

**Figure 1:**
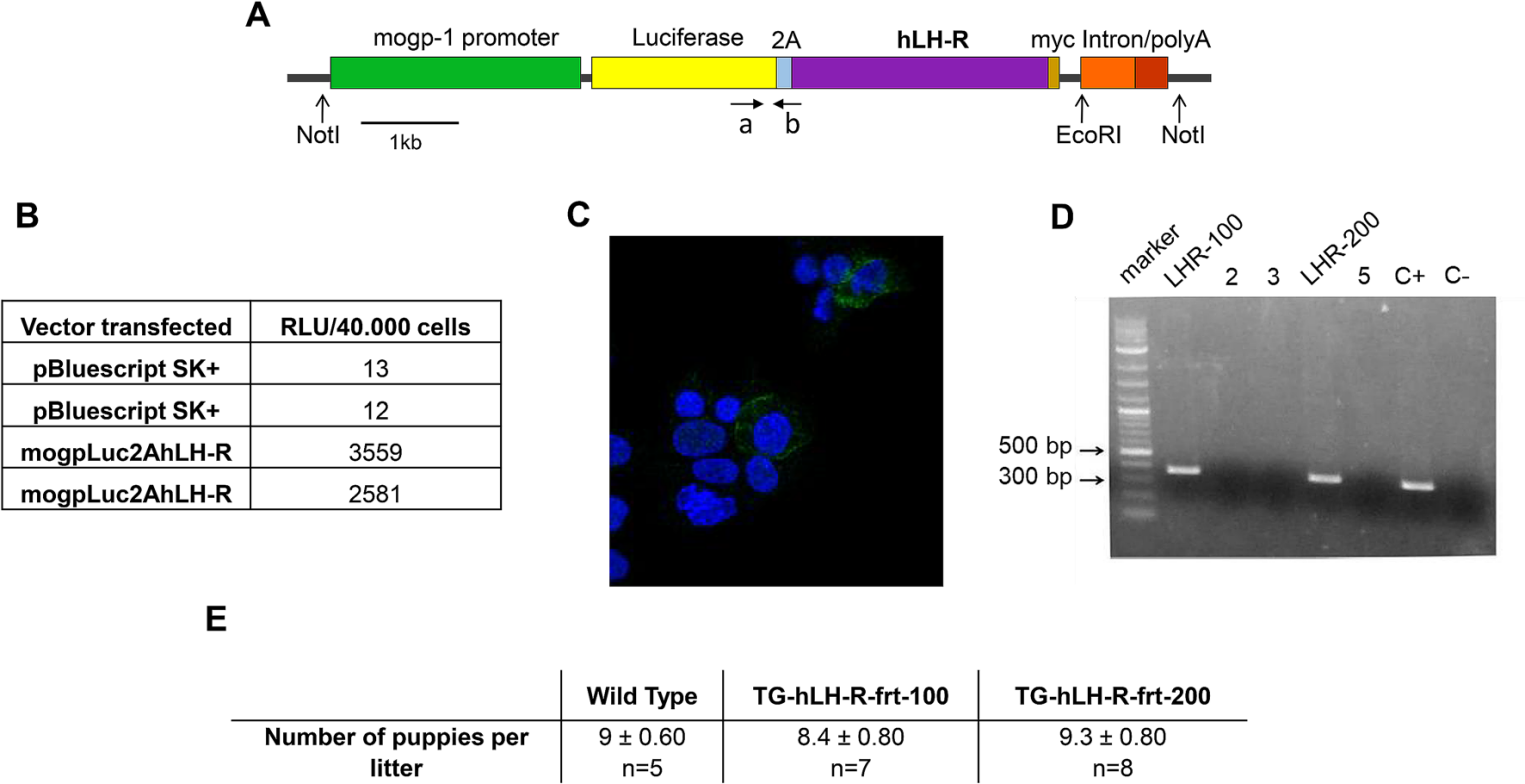
Generation of LH-R (TG) transgenic mice. (**A**): Scheme of the construct used for the generation of mice overexpressing LH-R. The LH-R and the Luciferase cDNA sequences are inserted in frame, separated by a specific viral sequence (2A sequence), allowing the production of the two single proteins in an equimolar manner. The unique site of digestion for Southern blot analysis (EcoRI) is reported. The primers used for the screening of TG mice mapping on the cDNA are indicated as a and b. (**B**): Luminescence data obtained after transfection of Hec1A cells with the pBluescript SK(+) vector containing the mogpLuc2AhLH-R transgenic construct or with an empty vector. The results of two different transfections are shown. Luciferine undergoes a luciferase-catalysed oxidation resulting in an excited state that emits upon decaying to its ground state. The resulting sample light output is measured by using a current-measuring luminometer whose output is expressed as arbitrary light units, usually referred to as “Relative Light Units” (RLU). (**C**): Confocal microscope images showing the presence of LH-R on the membranes (green spots) of Hec1A cells transfected as in (**B**). Staining with primary Ab anti-myc (1:100) and secondary Ab-Alexa 488 (1:500). The nuclei are counterstained with DAPI. (**D**): End point PCR on DNA extracted from tails of different puppies. Bands of the expected molecular weight (350 bp) are evident in two mice: lane 1 (LHR-100) and lane 4 (LHR-200); C+ is the vector that serves as positive control; C- is the negative control. (**E**): Summary table of pregnancy frequency: the mean number ± SEM of mice for each litter and the number of litters (n) for each mouse line (transgenic and WT) is reported.

To generate TG mice overexpressing hLH-R in the female reproductive tract (TG-hLH-R-frt mice), we first produced a transgene in which the human LH-R cDNA was put under the control of the oviduct-specific glycoprotein (OGP) mouse promoter (*mogp-1*). OGP is a member of the chitinase protein family normally secreted by non-ciliated epithelial cells of the oviduct and its promoter was previously used to drive the expression of the SV40 large-T antigen in the female reproductive tract (oviduct, ovary, uterus, including endometrium, and vagina)^18^. We used a tissue-specific promoter, even at the expense of a high expression of the transgene, to better mimic the expression levels reached by the LH-R encoding gene which occur in the human setting^18^. To detect even low expression of the transgene, we inserted the luciferase reporter gene (Luc upstream to hLH-R cDNA, and included the 2A peptide which allows the co-translational cleavage of proteins produced by a unique cDNA^19^, to drive an equimolar expression of the hLH-R cDNA and of the luciferase gene. A myc tag (c-myc) was inserted at the 3′-terminus of the hLH-R cDNA to allow easier detection of the encoded LH-R protein in tissues of TC mice. The characteristics of the final construct cloned in pBluescript SK(+) vector (mogpLuc2AhLH-R ) are shown in Fig. 1A.

The proper functioning of the transgene construct was tested transfecting it into Hec1A EC cells, where the OGP promoter is known to be active^18^, and evaluating the light emitted by the bioluminescent Luciferine. Hec1A cells transfected with the mogpLuc2AhLH-R vector showed a higher amount of Relative Light Units (RLU) compared to that of cells transfected with the pBluescript SK (+) empty vector (Fig. 1B). The proper expression of hLH-R on the plasma membrane of Hec1A cells transfected with the mogpLuc2AhLH-R construct was confirmed by IF, using a specific antibody against c-myc (Fig. 1C).

The mogpLuc2AhLH-R transgene, devoid of the plasmid backbone, was microinjected into the male pro-nucleus of mouse zygotes and the resulting puppies were screened by PCR analysis on tail DNA as detailed in “Materials and methods” section. Two out of five puppies turned out to be positive at the screening, one female and one male (Fig. 1D). Both mice turned out to be able to correctly transmit the transgene to their offspring, hence generating two TG lines: FVB-Tg(MOGP-hLH-R)100 and FVB-Tg(MOGP-hLH-R)200, hereafter abbreviated TG-hLH-R-frt-100 and TG-hLH-R-frt-200, respectively. Mice of the two transgenic lines obtained from either founder were maintained in heterozygosity in FVB background. The transgene appeared to be integrated in a head-to-tail tandem array with a higher copy number in the TG-hLH-R-frt-100 line compared to TG-hLH-R-frt-200 (Supplementary Figure S1). Both TG lines were fertile, with a mean number of born puppies similar to those obtained in wild type (WT) mice, with no changes in the number of litters over time (Fig. 1E; Supplementary Figure S2). Furthermore, the two TG lines had a similar number of follicles in the ovaries (Fig. 3), which is considered an indicator of intact fertility (see below). Anyway, the TG-hLH-R-frt-100 line was lost after 3 years.

**Figure 2:**
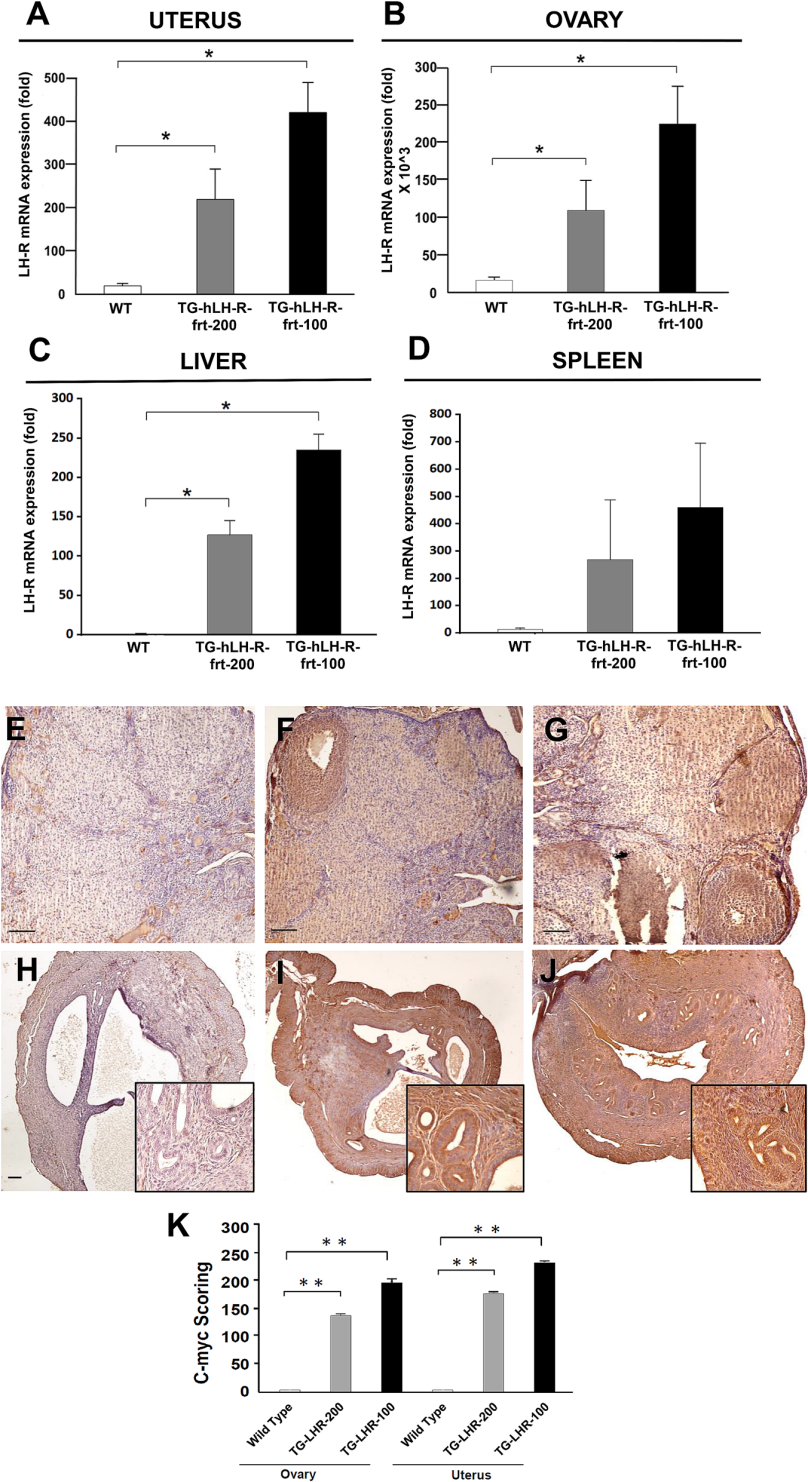
Evaluation of hLH-R expression in uteri and ovaries of TG mice. (**A**–**D**): Graphs representing LH-R mRNA expression values in different organs of TG-LH-R-frt-200 (grey bars), TG-LH-R-frt-100 (black bars) and WT mice (white bars). Folds values relative to each panel are reported below and *p*-values are in parentheses. (**A**) Uteri: 236 ± 62.8 folds in TG-LH-R-frt-200, 430 ± 67 folds in TG-LH-R-frt-100, 17.9 ± 8.5 in WT mice (*p* = 0.04 and *p* = 0.024, Student’s *T*-test). (**B**) Ovaries: 109,981 ± 29,772 folds in TG-LH-R-frt-200, 229,970 ± 46,071 folds in TG-LH-R-frt-100, 14,800 ± 3299 in WT mice (*p* = 0.042 and *p* = 0.017, Student’s *T*-test). (**C**) Liver: 127 ± 18.3 folds in TG-LH-R-frt-200, 235 ± 19.9 folds in TG-LH-R-frt-100, 1.3 ± 5.3 in WT mice (*p* = 0.02 and *p* = 0.007, Student’s T-test). (**D**) Spleen: 269 ± 217 folds in TG-LH-R-frt-200, 460 ± 235 folds in TG-LH-R-frt-100, 13.5 ± 4.3 in WT mice (*p* = 0.36 and *p* = 0.19, Student’s *T*-test). (**E**–**G**): Representative IHC pictures with anti c-myc antibody on ovaries of WT (**E**) TG-LH-R-frt-200 (**F**) and TG-LH-R-frt-100 mice (**G**). Nuclei are counterstained with hematoxylin. Bar = 200 μm. (**H**–**J**): Representative IHC pictures with anti c-myc antibody on uteri of WT (**H**), TG-LH-R-frt-200 (I) and TG LH-R-frt-100 mice (**J**). Nuclei are counterstained with hematoxylin. Bar = 200 μm. (**K**): Histogram summarizing c-myc scoring in the different samples (ovary and uterus) of WT and TG mice (sample size: 4 mice per group). Differences in c-myc expression between WT and TG mice are statistically significant: Ovary: WT vs TG-LHR-frt-200 *p* = 0.007; WT vs TG-LHR-frt-100 *p* = 0.0025. Uterus: WT vs TG-LHR-frt-200 *p* = 0.004; WT vs TG-LHR-frt-100 *p* = 0.0038 (*p*-values: Student’s *T*-test). In the figure, TG-LH-R-frt-100 and TG LH-R-frt-200 are abbreviated TG-LHR-100 and TG-LHR-200, respectively.

The expression of the transgene was quantified by Quantitative Real Time (RQ-) PCR, determining the amount of hLH-R in the RNA extracted from different tissues of 3 months-old female mice. In agreement with what shown by Miyoshi for the mogp promoter^18^ and in the website (http://www.informatics.jax.org/marker/MGI:106661) for endogenous Ovgp1 expression, the transgene turned out to be highly expressed in the uterus and ovary, as well as ectopically expressed in liver and spleen of TG mice compared to WT animals (Fig. 2A–D; raw data are in Supplementary Table S1). Nevertheless, no gross phenotypic alterations (such as hepato-splenomegaly, jaundice etc.) which could be related to such ectopic expression emerged. At difference from the other organs, in the ovary we found a significant basal expression of hLH-R (by RQ-PCR), possibly due to the partial overlap of the primers used for RQ-PCR with the mouse LH-R sequence. The expression of the hLH-R protein encoded by the transgene was confirmed both in the ovaries and in the uteri of either TG lines by IHC using anti-c-myc antibodies (Fig. 2E–J). The analysis of the IHC score (i.e. the product between the intensity and the percentage of positive cells) showed a very high score in both the ovary and uterus of both TG lines (Fig. 2K).

### Morphological and immunohistochemical characterization of TG-hLH-R-frt mice

**Figure 3:**
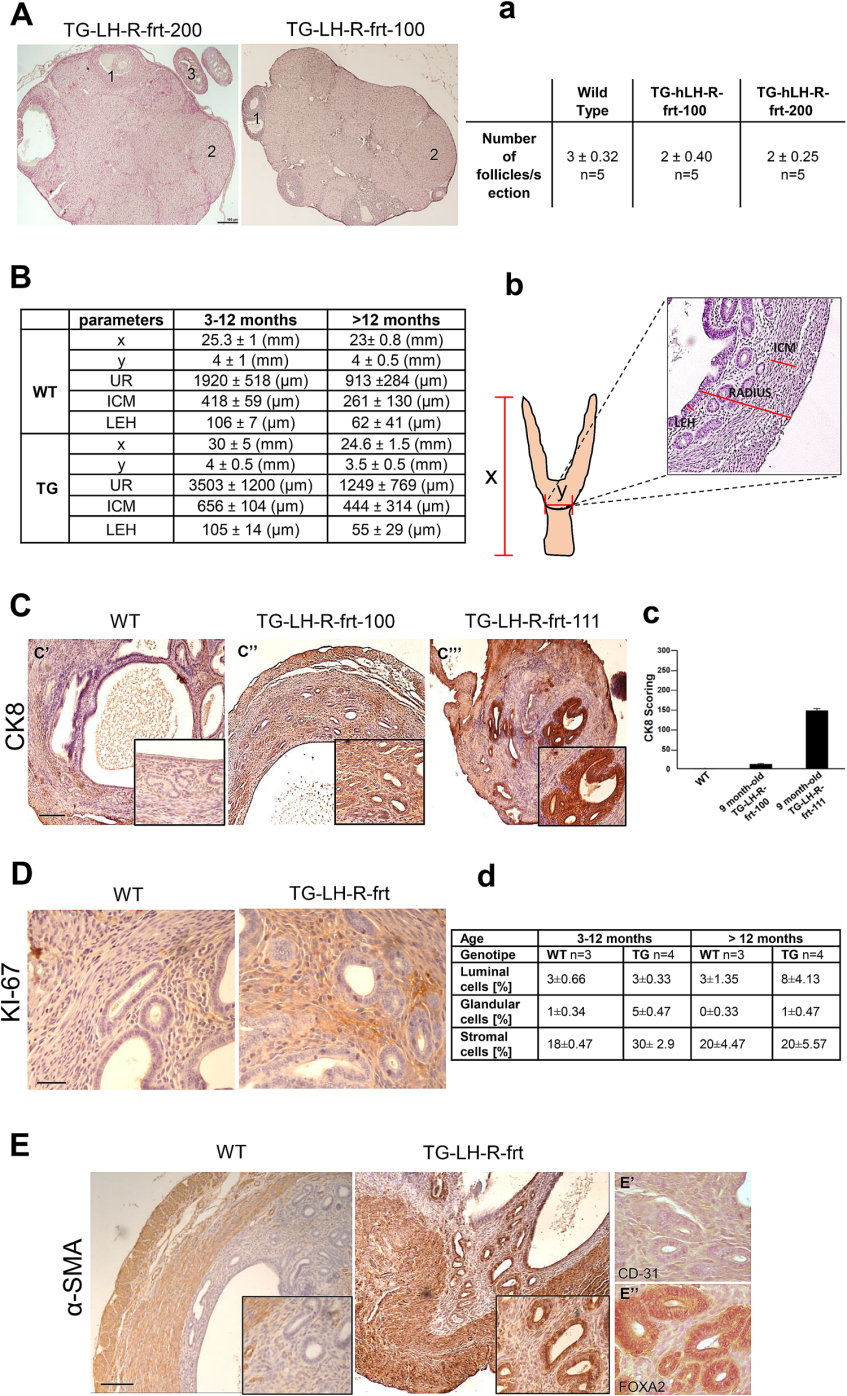
Characterization of the ovaries and uteri of transgenic mice. (**A**): H&E staining on representative ovary samples of TG-LHR-frt-200 mouse (left panel) and TG-LHR-frt-100 mouse (right panel). The ovaries do not show any histological alterations: antral follicles (1), corpus luteum (2) and the oviduct (3) are indeed still visible. Bar = 100 μm. (a): Resuming table in which the mean number ± SEM of antral and atretic follicles observed in each ovary slide is reported. We evaluated 5 mice per group (n = 5), 5 slides per mouse. (**B**): Table summarizing the characterization of the uteri of TG and WT mice in terms of size (x and y axes) and morphometry parameters (UR, ICM and LEH). Mice 3-12 months old: UR: TG vs WT *p* = 0.43; ICM: TG vs WT *p* = 0.21; LEH: TG vs WT *p* = 0.96. Mice > 12 months old: UR: TG vs WT *p* = 0.72; ICM: TG vs WT *p* = 0.64; LEH: TG vs WT *p* = 0.87. Values are shown as mean ± SEM. (**b**): The uterus longitudinal axis (x) and the transversal axis (y) of TG (of both the TG lines) and WT mice are reported. In the box a detail of a representative H&E staining showing UR, ICM and LEH is reported. (**C**): Representative IHC pictures of the uteri of WT and transgenic mice labelled with CK-8: (C’): 13 months-old WT mouse negative to cytokeratin 8; (C’’): 9 months-old TG-LH-R-frt-100, weakly positive; (C’’’): 9 month-old TG-LH-R-frt-111 (belonging to the TG-LH-R-frt-100 line) strongly positive to CK-8. Nuclei are counterstained with hematoxylin. Bar = 200 μm (**c**): Histogram summarizing CK-8 scoring in the different uteri samples (WT, TG-LH-R-frt-100 and TG-LH-R-frt-111). Sample size: 6 mice per group. (**D**): Representative IHC pictures of the uteri of WT and transgenic mice labelled with anti Ki67 antibody. Left panel: WT mouse. Right panel: TG-LH-R-frt mouse. Nuclei are counterstained with hematoxylin. Bar = 200 μm (**d**): Table summarizing Ki67 scoring in the different samples. The mean percentage of labeled nuclei evaluated in three different areas is reported. Mice are divided into two groups of age: 3-12 months old and older than 12 months. Statistically significant differences are observed between WT vs TG animals in glandular cells (TG vs WT *p* = 0.008, Fisher’s exact test) and stromal cells (TG vs WT *p* = 0.015, Fisher’s exact test) of 3-12 months-old mice. Sample size: 8 TG-LH-R-frt mice, 6 WT. 4 TG and 3 WT mice for each group of age. (**E**): Representative IHC pictures of the uteri of WT and TG mice labelled with anti α-sma antibody. Left panel: WT mouse. Right panel: TG-LH-R-frt mouse (of both the TG lines). (E’): Uterine slice of TG-LH-R-frt mouse stained with CD31 antibody: the staining is not present in the gland. (E’’): Uterine slice of TG-LH-R-frt mouse stained with FOXA2 antibody: only the glands are positive. Nuclei are counterstained with hematoxylin. Bar = 200 μm.

Based on the above expression data, we performed a morphological characterization of both the ovaries and the uteri of TG (from either transgenic lines) and WT mice at different ages: young (3–12 months) and old (> 12 months) aged mice. We did not detect any gross morphological or histological alteration in the ovaries of mice from either TG lines at any ages (representative pictures of > 12 months mice are in Fig. 3A; representative pictures of < 12 months mice are in Supplementary Figure S3). In particular, the two TG lines had a similar number of follicles in the ovaries (Fig. 3a), which indicates the preservation of ovulation capacity, hence suggesting the maintenance of proper fertility. We then analyzed the uteri from mice of either TG lines, quantifying uterine morphometry taking into account: the longitudinal and transversal uterus lengths (“Y” and “X” values in Fig. 3b, respectively), the uterine radius (UR), the inner circular muscle (ICM) and the height of the luminal epithelium (LEH) (Fig. 3B, b) as in Wood et al.^20^. While no differences in longitudinal and transversal uterus lengths, or in LEH emerged between TG and WT controls at young ages, the uteri of young TG mice (of either lines) showed an increased (although not statistically significant) mean UR and thickness of the ICM, compared to WT mice (Fig. 3B). TG and WT mice older than 12 months had similar UR and LEAH values, while the mean ICM thickness was higher in TG mice, although not statistically significant (Fig. 3B and Supplementary Figure S4). Two mice older than 12 months belonging to the TG line with the greater expression of the transgene (i.e. TG-hLH-R-frt-100) showed an increased (although not statistically significant) size of the uteri internal cavity, compared to age matched WT mice (Supplementary Figure S5). Because of the similarity of uterine features in either TG lines, we used these lines interchangeably hereinafter in our studies.

The endometrial layer in the uteri of TG mice was then better characterized by IHC analysis, evaluating Ki67 staining, to determine the extent of cell proliferation, cytokeratin 8 (CK-8, an epithelial cell-specific marker) and α-smooth-muscle actin (α-sma, a marker of stromal cells), to assess the state of cell differentiation^21^. Compared to WT mice, 33% (2 out of 6) of TG-hLH-R-frt mice were positive to CK-8 (Fig. 3C, c). The glandular epithelial and stromal cells of 6 months-old TG mice showed a statistically significant higher percentage of Ki67-positive cells, compared to WT animals (Fig. 3D, d). An increased Ki67 staining was observed in the luminal epithelial cells of TG mice older than 12 months, while it was no more evident in the stroma and glandular epithelium. Furthermore, the uteri of all (8 in total, randomly chosen) TG mice of both age groups (3–12 and > 12 months) showed a positive α-sma staining muscle and glandular epithelial cells (Fig. 3E, panels on the right). The glandular nature of α-sma positive structures was confirmed by their positivity to FOXA2 and negativity to CD31 staining (Fig. 3E′,E″). On the contrary, WT mice showed a significant staining in smooth muscle cells and a scanty signal in blood vessels (Fig. 3E, panel on the left).

Overall, IHC data corroborated morphological results, indicating the occurrence of epithelial hyperplasia and stromal trans-differentiation of epithelial cells, in the uteri of transgenic mice.

### Transcriptomic characterization of the uteri of TG-hLH-R-frt mice

Since the uterus was apparently the main organ affected by LH-R over expression, we studied in detail this organ, performing a whole transcriptomic analysis of the uteri of two young (6-months old) TG (belonging to the TG-hLH-R-frt-200 mouse line) and two age-matched WT mice. Applying the modified *p* value (modified Fisher Exact *p *value) less than 0.01 as threshold, we obtained a list of 386 differentially expressed (DE) genes between TG and WT mice: 115 upregulated and 271 downregulated (Supplementary file excel 1).

**Figure 4:**
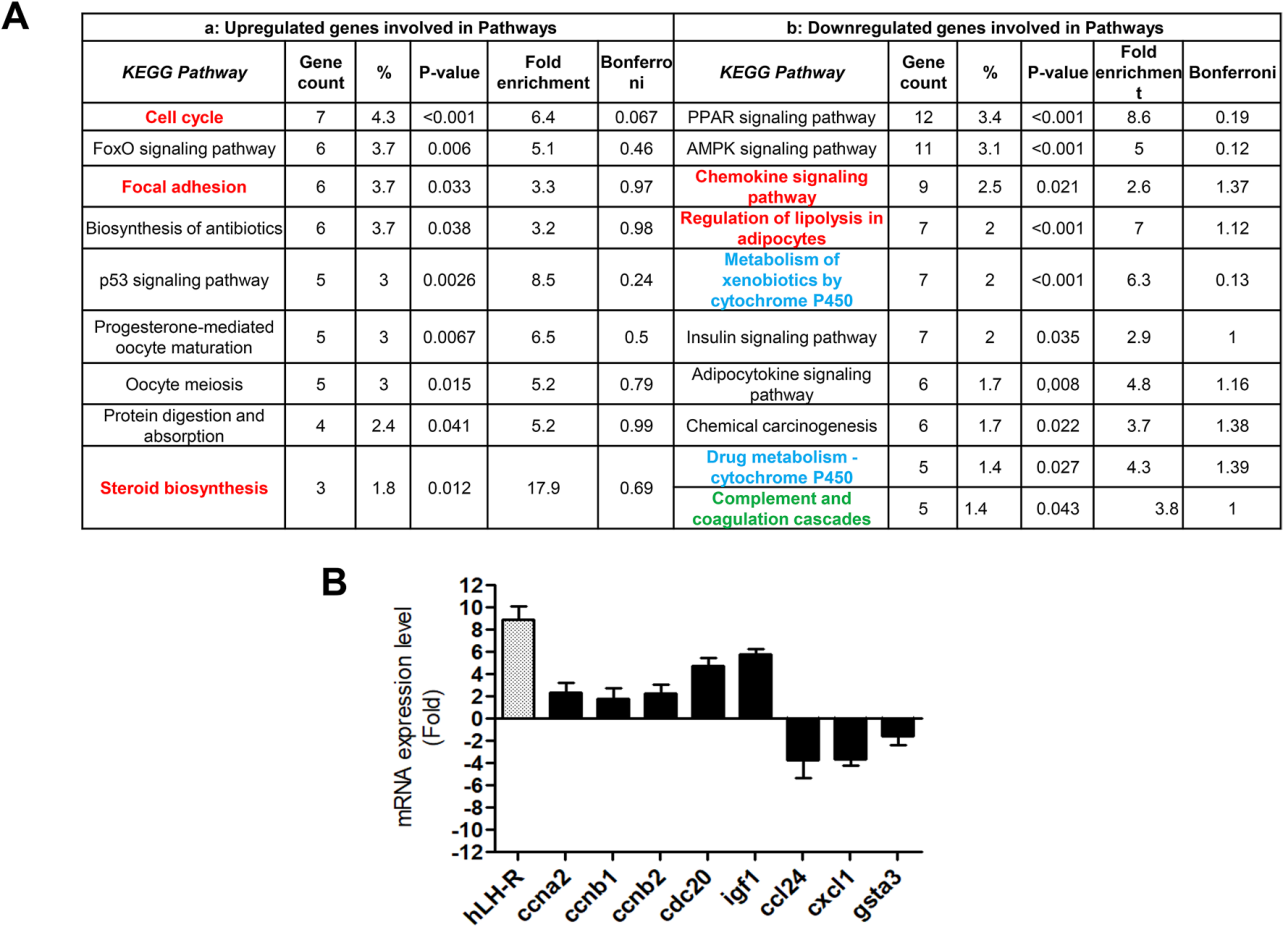
**A:** Pathways enrichment analysis of upregulated (**a**) and downregulated (**b**) genes, using *p*-values < 0.05 as threshold. The transcriptomic analysis was performed on uteri of two 6-months old TG mice (belonging to the TG-hLH-R-frt-200 mouse line) and WT mice. Table shows the number and the percentage of genes involved in each pathway, with the relative fold enrichment. For this analysis the Bonferroni correction was applied. (**B**): Bar-graph of the mRNA fold increase of 8 genes (excluding hLH-R) showing alterations between TG and WT mice in the microarray data. Data are reported as log2 of 2^-DDCt^. The Log2 fold change values from microarray data and Log2 fold change values from RQ-PCR data are used to perform Pearson Correlation Test. *p* = 0.0032 (two-tailed *t*-test; n = 4, 2 WT vs 2 TG). R square = 0.85 (Pearson correlation coefficient).

The Functional annotation analysis (FAA) performed using the NIH-DAVID database, revealed that the majority of the upregulated genes belong to four functional categories, e.g. “terms”: ATP binding, microtubule, protease inhibitor and Ubl conjugation. We also analyzed the DE genes according to their possible role in known pathways (Fig. 4A). We found upregulated genes (Fig. 4A and Supplementary Table S2) involved in the p53 pathway, as well as in cell cycle pathway, such as cyclins (*Ccna2*,* Ccnb1*,* Ccnb2*) and members of the cyclin-dependent kinases family (such as *Cdk1*). Other upregulated genes belong to biological processes such as: progesterone-mediated oocyte maturation, steroid biosynthesis and focal adhesion. The FAA of down regulated genes (Fig. 4A and Supplementary Table S3) showed that 82 (30%) genes were related to either the AMPK (i.e. protein phosphatase 2A gene, *PPA2*) and the PPAR signaling pathways. Interestingly, some of the downregulated genes codify proteins involved in protective mechanisms against damage or in toxic compounds metabolism: the metabolism of xenobiotics by P450, drug metabolism-P450 cytochrome, and chemical carcinogenesis. In addition, some downregulated genes belong to the chemokine signaling, such as *Cxcl1*, *Cxcl10*, *Cxcl15*, *Cxcr2*, *Ccl17*, *Ccl24* and *Ccl4*. Eight of the genes belonging to the most DE pathways (cell cycle, FoxO signaling pathways, focal adhesion, p53 signaling pathways, chemokine signaling pathways, metabolism of xenobiotics by cytochrome P450 and damage protection from chemical carcinogenesis) of TG-hLH-R-frt mice were then validated by RQ-PCR (Fig. 4B). A statistically significant correlation was obtained between Log2 fold change values from microarray data and Log2 fold change values from RQ-PCR (*p* = 0.0032, *p* value by two-tailed *t* test), with a R square = 0.85 (R = Pearson correlation coefficient). In the same samples a high expression of the h-LH-R transgene, evaluated by RQ-PCR with primers that recognize both human and mouse orthologs, also emerged, further confirming dataset validity (Fig. 4B).

Overall, transcriptomic data further support that LH-R overexpression in the uterus dysregulates the expression of genes involved in cell proliferation, differentiation as well as damage protection and inflammation. This was also confirmed by comparing the transcriptomic data of our TG mice with FOXO1 uterine KO^22^ and progesterone (Wnt7acre/+mPgrALsL/+) overexpressing models^23^ (see the complete analysis in Supplementary Table S4), Some DE pathways (e.g. cell cycle, inflammation and cell–cell contact) turned out to be shared by the three TG mice (Fig. 4A, highlighted in red). The “small molecules biochemistry related pathway” turned out to be deregulated both in our TG model and in the progesterone overexpressing model (Fig. 4A, highlighted in blue). Pathways related to the occlusion of vessels and arteries were downregulated in the FoxO KO model, which is comparable with the downregulation of the complement and coagulation cascades pathways observed in our LH-R over-expressing mice (Fig. 4A, highlighted in green).

### Aged female TG-hLH-R-frt mice develop uterine tumor masses.

**Figure 5:**
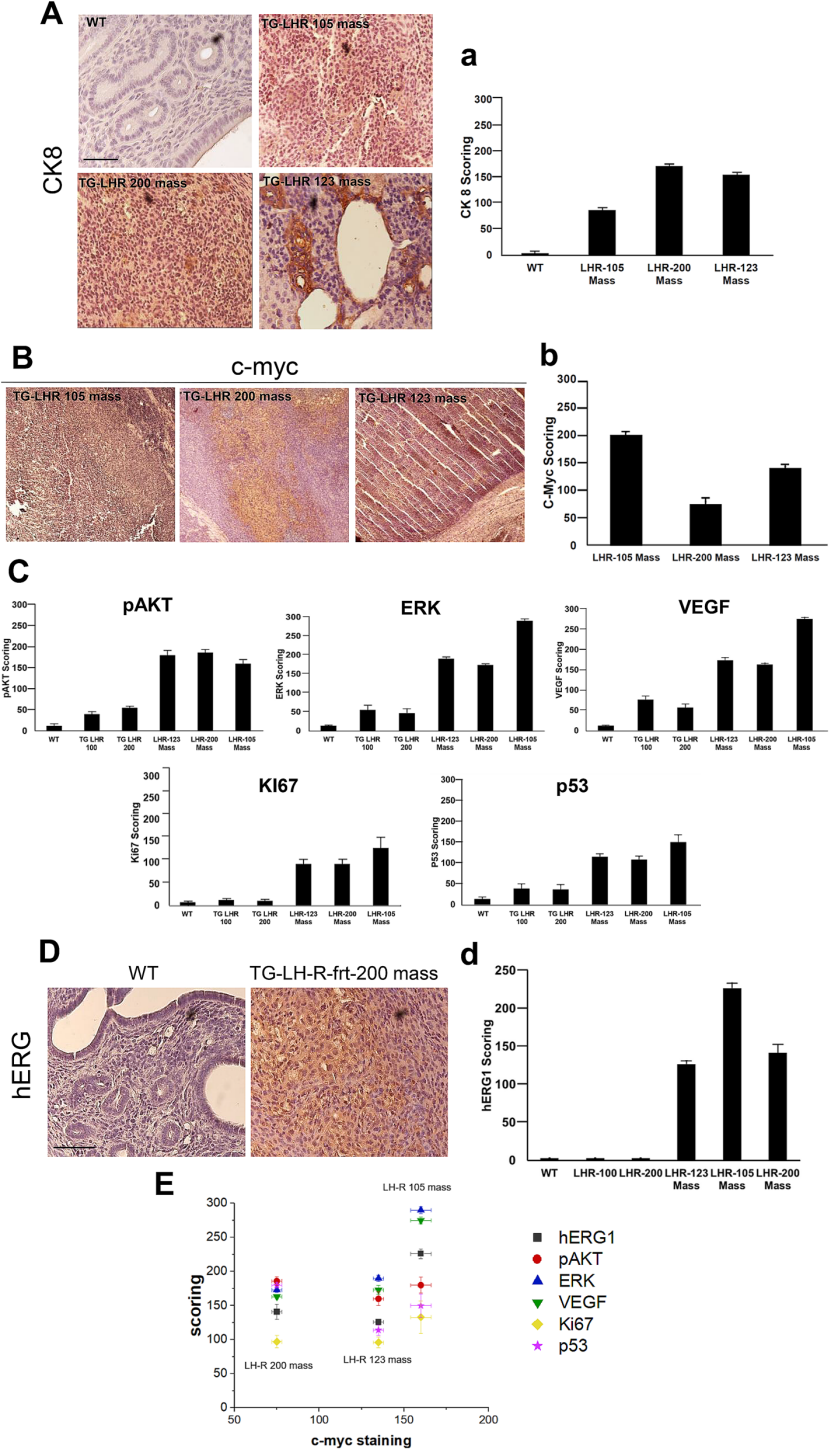
Pathological findings in aged female transgenic mice. **A:** IHC staining with anti-CK-8 antibody on masses derived from TG-LH-R-frt-105, TG-LH-R-frt-200 and TG-LH-R-frt-123. Nuclei are counterstained with hematoxylin. Bar = 200 μm **a:** histogram summarizing CK-8 scoring in the different tumor masses (TG-LH-R-frt-105 mass: 86 ± 4.5; TG-LH-R-frt-200 mass: 172 ± 4.4 and TG-LH-R-frt-123 mass: 155 ± 3; WT score = 0). **B:** Representative IHC pictures of masses derived from TG-LH-R-frt-105, TG-LH-R-frt-200 and TG-LH-R-frt-123 labelled with anti-c-myc antibody. Nuclei are counterstained with hematoxylin. **b:** Histogram summarizing c-myc scoring in the different tumor masses (TG-LH-R-frt-105, TG-LH-R-frt-200: 75 ± 3, TG-LH-R-frt-123: 105:160 ± 6). **C:** histograms summarizing pAKT. ERK, VEGF, Ki67 and p53 scoring in the different tumors (pAKT: WT: 12 ± 5; healthy TG-LH-R-frt-100 40 ± 7; healthy TG-LH-R-frt-200 55 ± 5; TG-LH-R-frt-123 mass: 160 ± 17.3; TG-LH-R-frt-200 mass: 186 ± 6.6; TG-LH-R-frt-105 mass: 180 ± 11.5. ERK: WT: 13 ± 2; healthy TG-LH-R-frt-100: 55 ± 13; healthy TG-LH-R-frt-200: 46 ± 12; TG-LH-R-frt-123 mass: 190 ± 5; TG-LH-R-frt-200 mass: 173 ± 3.5; TG-LH-R-frt-105 mass: 290 ± 5. VEGF: WT: 11 ± 1, healthy TG-LH-R-frt-100: 77 ± 9, healthy TG-LH-R-frt-200: 57 ± 8.8, TG-LH-R-frt-123 mass: 173 ± 7, TG-LH-R-frt-200 mass: 163 ± 3; TG-LH-R-frt-105 mass: 275 ± 5. Ki67: WT: 8 ± 1.5, healthy TG-LH-R-frt-100: 13 ± 3.7, healthy TG-LH-R-frt-200: 11 ± 3.8, TG-LH-R-frt-123 mass: 96.6 ± 9, TG-LH-R-frt-200 mass: 96.6 ± 9; TG-LH-R-frt-105 mass: 133 ± 24. P53: WT: 13 ± 4, healthy TG-LH-R-frt-100: 35 ± 10, healthy TG-LH-R-frt-200: 33 ± 11, TG-LH-R-frt-123 mass: 115 ± 7.5, TG-LH-R-frt-200 mass: 108 ± 7.5; TG-LH-R-frt-105 mass: 150 ± 17.3). **D:** Representative IHC picture using image anti-hERG1 antibody on the uterus of WT mouse and the mass derived from TG-LH-R-frt-200. The expression of hERG1 and c-myc is evaluated in adjacent tumor sections. Nuclei are counterstained with hematoxylin. Bar = 200 μm. **d:** Histogram summarizing hERG1 score quantification in the different samples (WT: 0, healthy TG-LH-R-frt-100 mice: 0, healthy TG-LH-R-frt-200 mice: 0, TG-LH-R-frt-123 mass: 226 ± 7, TG-LH-R-frt-105 mass: 141 ± 11 and TG-LH-R-frt-200 mass: 126 ± 5). E: Scatter plot of hERG1, pAKT, ERK and VEGF in the three different masses. Values are means ±SEM. c-myc vs hERG1: *p* = 0.014, R=0.8991; c-myc vs pAKT: *p* = 0.013, R = 0.9048; c-myc vs ERK: *p* = 0.0017, R = 0.9661; c-myc vs VEGF: *p* = 0.005, R = 0.9431; c-myc vs Ki67: *p* = 0.007, R = 0.9275; c-myc vs p53: *p* = 0.002, R = 0.9624 (*p*-values are evaluated by Student’s t test; R = Pearson Correlation Coefficient).

In two aged (17-months-old) female TG-hLH-R-frt mice from either TG lines (TG-hLH-R-frt-105 and TG-hLH-R-frt-200) we observed signs of suffering such as slow motility and rough fur. Animals were then euthanized and the abdominal cavity examined, showing the presence of large masses (3 cm^3^ in the TG-hLH-R-frt-105 mouse and 2.8 cm^3^ in the TG-hLH-R-frt-200 mouse) in the lower abdomen, at the uterine level. Moreover, one 17-months-old mouse, belonging to the line LH-R-100 (TG-hLH-R-frt-123) showed a smaller-size mass (45 mm^3^) in the lower-third of the left uterine horn when randomly sacrificed for the routine characterization analysis (Supplementary Figure S6). The histology of the large-sized masses of TG-hLH-R-frt-105 and TG-hLH-R-frt-200 mice showed a poorly differentiated tissue and the loss of the typical uterine architecture (Supplementary Figure S6A, B). Both masses were composed of cells with big and highly basophilic nuclei at the H&E staining. The small mass of TG-hLH-R-frt-123 showed a loss of the normal uterine architecture, although the inner circular muscle layer was still visible (Supplementary Figure S6C). Overall, 3 out of 9 (33%) female TG mice who reached an age > 17 months showed masses at the uterine level. All the masses positively stained with CK-8, indicating their origin from endometrial epithelial cells (Fig. 5A). The mass derived from the TG-hLH-R-frt-105 showed a slight diffuse staining, while that of the TG-hLH-R-frt-200 showed a more intense and homogeneous staining (Fig. 5A). In the mass derived from the mouse TG-hLH-R-frt-123 the staining was localized in areas resembling glandular structures, while was absent in surrounding tissues (Fig. 5A). Applying the scoring system described in "Materials and methods" section, all the tumor masses showed a higher mean score compared to WT (Fig. 5a). As expected, all the tumor masses positively stained for c-myc (Fig. 5B) with high scores (Fig. 5b). This indicates that hLH-R is indeed expressed in the tumor masses.

Overall, the histology of the masses arising in aged transgenic females is suggestive of a malignant endometrial tumor mass, i.e. an EC.

We then performed a transcriptomic analysis of the tumor mass originated in the TG-hLH-R-frt-200 mouse, comparing the gene expression profile (GEP) of the tumor mass with that of the uterus of an aged-matched TG-hLH-R-frt mouse (belonging to the same TG-hLH-R-frt-200 line), but with no evidence of tumor mass. The modified *p* value less than 0.01 was used as threshold. Than a gene was assumed to be DE when the fold change (log2 fold change) was ≥ 2. Applying these thresholds, a list of 1701 DE genes was obtained, 718 upregulated and 983 downregulated. The FAA led to identify the most altered biological processes: DE genes were significantly associated to 20 terms with a false discovery rate (FDR) *p* value < 0.05 (Supplementary Table S5 and S6). The most significant terms associated to upregulated genes were related to the membrane term, as well as to terms related to inflammatory response, positive regulation of ERK1 and ERK2 cascade, signal transduction, chemokine-mediated signaling pathway and positive regulation of angiogenesis (Table 1a). On the other hand, downregulated genes were associated to terms related to epithelial cell differentiation, positive regulation of cell migration, positive regulation of epithelial cell proliferation, focal adhesion, positive regulation of angiogenesis, apical plasma membrane and calcium ion binding (Table 1b). Notably, we detected the downregulation of two genes encoding proteins essential for the establishment of cell–cell contacts: *CTNNAL1* gene (α-catenin) and *CDH2* gene (N-cadherin) (Supplementary Table S6), and of the genes encoding the transforming growth factor beta receptor III (*TGFBR3*), *LAMC2* (laminin-γ2) and *MSX1* (Msh homeobox) (see the genes highlighted in bold in Supplementary Table S6), which are known to be downregulated in human EC^23,24^. The most relevant DE genes are indicated in the “most relevant genes” column in Table 1. Furthermore, we validated the identified signature of the tumor mass of TG-hLH-R-frt-200 mouse comparing it with the gene expression profile of other ECs obtained from publicly available datasets deposited into the GEO databases. We chose the datasets E‐MTAB‐2532, E‐GEOD‐56026, GSE32507, GSE24537, E‐GEOD‐23518, E‐GEOD‐17025, E‐GEOD‐2109, analysed in O’Mara 2016 and the datasets GSE17025, GSE63678 and GSE35794, analysed in Liu 2018^26,27^. Two out of the 1701 DE genes found in the tumor mass of the TG-hLH-R-frt-200 mouse matched with the 10 more downregulated genes identified by Liu 2018 in EC, including Tgfbr3. When compared with the 145 more deregulated genes from O’Mara 2016, 11 commonly deregulated genes emerged, including Sox17 and Esr1 (the whole analysis is shown in Supplementary Table S9, and the most relevant genes in common with the above datasets are highlighted in red in Table 1). Overall, the transcriptomic analysis of the masses arise in aged TG females supports the conclusion that they are endometrial tumor masses.

**Table 1:** Upregulated and downregulated terms between the tumor mass derived from the LH-R-frt-200 mouse and the healthy TG-LH-R-frt mouse.

Category	Term	Most relevant genes	Count	FDR
**(a) Upregulated terms**				
GOTERM_BP_DIRECT	Inflammatory response		57	< 0.001
GOTERM_CC_DIRECT	MEMBRANE	Ryr1	314	< 0.001
GOTERM_BP_DIRECT	Positive regulation of ERK1 and ERK2 cascade		20	0.001
GOTERM_BP_DIRECT	Chemokine-mediated signaling pathway		11	0.003
GOTERM_BP_DIRECT	Cellular response to interferon-gamma		12	0.003
GOTERM_BP_DIRECT	Signal transduction		62	0.009
GOTERM_BP_DIRECT	Positive regulation of GTPase activity		16	0.017
GOTERM_MF_DIRECT	Cytokine receptor activity		9	0.034
GOTERM_BP_DIRECT	Positive regulation of angiogenesis		14	0.041
**(b) Downregulated terms**				
GOTERM_CC_DIRECT	Extracellular exosome	Sytl1, Gcnt3, Tmc4, Tst, Tmprss2, Tgfbr3, Cdh2	209	< 0.001
GOTERM_BP_DIRECT	Epithelial cell differentiation	Ehf, Tst	14	0.002
GOTERM_CC_DIRECT	Apical plasma membrane	Tjp3, Marveld2, Cdh2	32	0.006
GOTERM_BP_DIRECT	Positive regulation of cell migration	Lamc2	24	0.008
GOTERM_BP_DIRECT	Positive regulation of transcription from RNA polymerase II promoter	Ehf, Sox17, Esr1, Hand2, Msx1	69	0.009
GOTERM_BP_DIRECT	Bicellular tight junction assembly	Marveld2	9	0.010
GOTERM_BP_DIRECT	Positive regulation of epithelial cell proliferation	Esr1,	14	0.012
GOTERM_CC_DIRECT	Focal adhesion		35	0.012
GOTERM_MF_DIRECT	Calcium ion binding	Sytl1, Cdh2	51	0.022
GOTERM_BP_DIRECT	Positive regulation of angiogenesis		17	0.035
GOTERM_CC_DIRECT	Cytoskeleton	Ctnnal1	71	0.049

Terms most significantly upregulated (a) and downregulated (b) between the tumor mass derived from the LH-R-frt-200 mouse and the healthy TG-LH-R-frt-200 mouse.

We further validated the upregulation of signaling pathways controlling cell proliferation and angiogenesis, through an IHC analysis of pAkt, ERK1, VEGF, Ki67 and p53. All the tumor masses overexpressed the above markers, and a statistically significant positive correlation emerged between c-myc and VEGF, ERK, pAKT, Ki67 and p53 score (Fig. 5C). Interestingly, the ERK and VEGF expression profile better matched the c-myc expression profile (i.e. the hLH-R overexpression) in the same tumor mass (Fig. 5C).

Among the DE genes, the “membrane” term contained the vast majority of the upregulated genes. Among them, we observed several genes encoding for ion channels (e.g. KCNK13, CACNA1F, TRPV2, P2RX4, P2RX7) and transporters (e.g. SLC2B, SLC 7A7, SLC 11A1, SLC 15A, ABCA1, ABCA9, ATP1A3, ATP13A2). Moreover, we performed an IHC analysis on the tumor endometrial masses arising in TG-LH-R-frt mice, using a hERG1 specific antibody, which also recognizes the mouse ERG1^27^. It emerged that while the uteri of either WT or TG did not express the potassium channel, all three tumor masses arising in TG showed a high level of expression of hERG1 (Fig. 5D, d). Interestingly, a statistically significant positive correlation emerged between c-myc Tag (which indicates the expression of the transgene) and hERG1 score (Fig. 5E) (*p* value: 0.014; R: 0.8991, Pearson Correlation Coefficient).

### Patients with endometrial cancer (EC) express LH-R: clinico-pathological correlations

Based on the above data and on the previously described expression of LH-R^10–12^ and Kcnh2^28^ in human primary ECs, we performed a transcriptomic analysis (applying RQ-PCR analysis) on a cohort of 126 patients with EC of various stages and grades (Raw data are in Supplementary Table S10). All the EC samples turned out to express the LH-R at high levels with a median value of 73.78 (folds). LH-R expression levels significantly associated with risk (high LH-R expression and low risk; *p* = 0.025) and myometrial invasion (high LH-R expression and myometrial infiltration less than 50% of myometrial depth; *p* = 0.014) (Table 2). In addition, most of the EC samples turned out to express the Kcnh2 at high levels with a median fold value of 79.6. Statistically significant correlations were found between Kcnh2 high expression and low FIGO stages (*p* = 0.01), low risk endometrial cancer (*p* = 0.019) and infiltration less than 50% myometrial depth (*p* = 0.036) (Table 2). Finally, LH-R expression significantly associated with the expression of Kcnh2 (*p* = 0.006, Fisher’s exact test; Spearman Index = 0.245).

**Table 2:** Association between LH-R and Kcnh2 mRNA expression and clinicopathological parameters calculated with Spearman method and *p* values associated to the Spearman index (Sp.index).

Variable category	LH-R up (%)	LH-R down (%)	Spearman correlation text		Kcnh2 up (%)	Kcnh2 down (%)	Spearman correlation text	
			Sp. index	*p* value			Sp. index	*p* value
**FIGO**								
IA,IB	56(52.8)	53(47.2)	0.046	0.61	59(54)	50(46)	− 0.228	**0.01**
II	5(62.5)	3(37.5)			4(50)	4(50)		
IIIA,IIIB,IIIC	4(55.5)	5(44.5)			1(11)	8(89)		
IV	0(0)	0(0)			0(0)	0(0)		
**Histotype**								
Endometrioid	61(54)	52(46)	0.049	0.59	58(51.4)	55(48.6)	0.150	0.095
Non Endometrioid	4(30.8)	9(69.2)			4(30.8)	9(69.2)		
**Grading**								
G1	23(65.7)	12(34.3)	− 0.158	0.082	23(65.7)	12(34.3)	− 0.172	0.057
G2	29(49.1)	30(50.9)			26(44)	33(56)		
G3	13(40.6)	19(59.4)			13(40.6)	19(59.4)		
**Risk**								
Low	41(62.1)	25(37.9)	− 0.200	**0.025**	38(57.5)	28(42.5)	− 0.213	**0.019**
High	24(40)	36(60)			23(38.3)	37(61.7)		
**Myometrial invasion**								
< 50%	45(62.5)	27(37.5)	− 0.224	**0.014**	42(58.3)	30(41.7)	− 0.192	**0.036**
> 50%	21(38.8)	33(61.2)			21(38.8)	33(61.2)		
**Kcnh2**								
Up	39(64)	22(36)	0.245	**0.006**				
Down	26(40)	39(60)						
**LH-R**								
Up					39(60)	26(40)	0.245	**0.006**
Down					22(36)	39(64)		

Significant correlations (*p* < 0.05) are highlighted in bold. Kcnh2 and LH-R expression were considered upregulated and downregulated (marked as up and down in the table) for values greater and lower than the median value respectively.

## Discussion

In the present study we generated TG mice over-expressing the human form of the LH-R encoding gene in the female reproductive tract (TG-hLH-R-frt mice), to study the role of LH-R mis- and over-expression in the initiation and progression of cancers of the female reproductive system, in particular of EC.

To drive the expression of the hLH-R in the female reproductive tract, as well as to better mimic the expression levels reached by the LH-R encoding gene occurring in the human setting^18^, we exploited the mogp-1 promoter. Furthermore, the 2A peptide^19^ was included into the construct to drive an equimolar expression of the hLH-R cDNA and of the luciferase gene, a device to monitor the proper expression of LH-R, which we exploited during the initial check of the appropriateness of the construct once transfected into Hec1A cells (see Fig. 1).

TG-hLH-R-frt mice were vital and displayed a normal fertility, at least for four generations. One of the two TG lines, the TG-hLH-R-frt-100 expressing the hLH-R mRNA at higher levels, however, experienced a decline in fertility after 3 years, and was lost afterwards.

A high expression of the transgene (witnessed by the expression of the hLH-R mRNA and by the immunohistochemical detection of the c-myc tag) occurred in the ovaries and the uteri of transgenic females, as expected on the bases of the reported expression of the *mogp-1* promoter^18^. An ectopic expression in the liver and spleen was also observed, which however did not produce any pathological phenotype. It is worth noting that ectopic expression of transgenes can often occur as a result of the position effect from random integration into the genome^29^. In addition, despite the very high hLH-R expression in the ovaries, we did not observe gross morphological or functional alteration in this organ, in particular no difference in the number of follicles, which could explain the lack of effects on fertility.

On the contrary, TG-hLH-R-frt females showed alterations in the proliferation and differentiation state of the endometrial layer, which were more evident in aged mice. Indeed, both epithelial, either glandular or luminal, and stromal cells of the uterine mucosa had a high Ki67 staining, and an aberrant expression of α-sma, which is normally expressed in myofibroblasts, but absent in the glandular epithelium^21^. Overall, signs of hyperplastic growth and trans-differentiation were evident in the uteri of TG-hLH-R-frt mice, starting from 3 months and were maintained afterwards (Supplementary Table S7).

A whole transcriptomic analysis, performed on the uteri of young (6 months old) mice showed the deregulation of several genes involved in cell proliferation, differentiation as well as damage protection and inflammation. In particular, a high expression of genes involved in the p53 pathway, as well as in the regulation of the cell cycle and proliferation emerged. The upregulation of the p53 pathway related genes merits attention, since mutations of p53 are common in Type II EC, while are late events and hence apparently only contribute to progression of Type I ECs^30^. The upregulation of cyclins together with cyclin-dependent kinases family are suggestive of a possible dysregulation in cell cycle, control of cell death and DNA damage repair, hence predisposing cells to malignant transformation^31^. Between the downregulated pathways, AMPK signaling pathways is noteworthy. This pathway is involved in regulating growth and reprogramming metabolism and includes the downregulated protein phosphatase 2A gene (*PPA2*), which is one of the four major Ser/Thr phosphatases, and it is implicated in the negative control of cell growth and division^32^. Another downregulated pathway is the PPAR signaling pathway. PPAR pathway plays essential roles in the regulation of cellular differentiation, development, and metabolism^33^. Moreover, it was observed a lower expression of genes involved in protective mechanisms against toxic compounds (such as cytochrome P450 family genes) and genes belonging to chemokine signaling, suggesting a reduced immune response. Both the inability of metabolizing toxic compounds and the impairment of the native and adaptive immune system are signs of vulnerability and predisposition to cancer. The comparison between TG-hLH-R-frt mice with FOXO1 uterine KO and progesterone overexpressing mice, evidenced commonly altered processes, such as the deregulation of pathways related to the inflammatory response process and cell survival, thus reinforcing the suggestion of a role of LH-R in the FoxO and progesterone signaling pathways and thus in the development of ECs.

Considered together, all the dysregulated pathways emerging from the transcriptomic analysis are suggestive of an increased cell proliferation, of an alteration of epithelial differentiation accompanied by a lower ability of the cells to respond to damage, including cancerogenic insults.

Consistently, 33% of old (> 17 months) TG-hLH-R-frt female mice spontaneously developed masses at the uterine level, which resemble human ECs (see Supplementary Table S5). Indeed, these masses showed a poorly differentiated tissue and the loss of the typical uterine architecture, and were then interpreted by us as ECs. This was confirmed by the high expression of CK-8 as well as by the results of transcriptomic analysis. EC is characterized by many genetic and molecular alterations, in particular those concerning proteins responsible for signal transduction and cell adhesion^34^. Consistently, we found the downregulation of N-cadherin (*CDH2*) and α-catenin (*CTNNA1*) genes in the tumor mass of TG-hLH-R-frt-200 mouse, according to what occurs in human ECs^35^. In particular, both cadherins and catenins are closely involved in cell adhesion, and α-catenin is involved in the epithelial-mesenchymal transition (EMT). A decreased expression of epithelial markers (such as E-cadherin and α-catenin) has been detected in human EC tissue^36^. Hence, the dysregulation of both cadherins and catenins represents a central aspect in the aggressive behavior of EC. Furthermore, in the tumor masses arising in TG mice, we detected the downregulation of *LAMC2* and *MSX1* (laminin subunit gamma 2- and MSH homeobox 1-encoding genes, respectively), which are also downregulated in human poorly differentiated EC^24^. Finally, the transcriptomic analysis performed in the tumor masses arising in the uteri of old female TG mice, showed the dysregulation of genes encoding angiogenic factors, such as the vascular endothelial growth factor (VEGF), frequently dysregulated in ECs^37^. We also validated the identified signature of the tumor mass of TG-hLH-R-frt-200 mouse comparing it with the GEP of other endometrial cancer obtained from publicly available datasets deposited into the GEO database. Interestingly, some common deregulated genes emerged. For example, the downregulation Tgfbr3, whose reduced expression has been demonstrated in multiple types of human cancer including breast, prostate, ovarian, pancreatic, non-small cell lung cancer and renal cell carcinoma^38^. Among other deregulated genes, the downregulation of Sox17, identified as tumor suppressor in endometrial cancer^39^ and the deregulation of Esr1, involved in epithelial-mesenchymal transition (EMT) and considered as prognostic marker for endometrial cancer^40^, which confirm our results merit further considerations. This comparison with other endometrial cancer gene signatures validates the clinical relevance of gene expression profile here identified.

We confirmed some of the deregulated pathways emerging from the transcriptomic analysis by IHC. In particular, a statistically significant positive correlation emerged between the expression of hLH-R (witnessed by c-myc expression) and VEGF, ERK, pAKT, Ki67 and p53 staining. The upregulation of pAkt is particularly interesting, since it confirms what described in human Type I ECs, where the most common genetic mutations are detected in the Pten (phosphatase and tensin homolog) gene^41^, and what shown in Pten -/- mice^21^. Finally, an interesting correlation also emerged between hLH-R expression and the overexpression of the hERG1 potassium channel in the tumor masses, confirming data obtained in several human cancers^28^, including EC^25^. The clinical relevance of LH-R over-expression in EC and the correlation between LH-R and Kcnh2 (hERG1) mRNA expression in primary human ECs was strengthen evaluating LH-R and Kcnh2 mRNA expression by RQ-PCR in a cohort of 126 human EC specimens. A significant positive correlation between the expression level of these two genes emerged. Moreover, significant correlations were found between the high expression of LH-R and Kcnh2 and low-risk EC subgroup and between the high expression of the two genes and invasion less than 50% myometrial depth.

Taken together, these data corroborate the main finding obtained in the present work, i.e. the causative role of LH-R over expression in the development and malignancy of EC. In particular, LH-R overexpression apparently leads to an increased probability to develop spontaneous endometrial tumor masses. Whether LH-R overexpression could act making mice more susceptible to external carcinogens, including hormones, or could trigger per se the cancerogenic process, by activating pro-neoplastic signaling pathways, remains a matter of fact, to be further deciphered. In this scenario, taking into account the association between LH-R and hERG1 expression, we could hypothesize that plasma membrane receptors (in our case LH-R) and hERG1 cooperate in triggering intracellular signaling pathways in EC as previously shown in several cancer types^42^. Besides speculations, the targeting the of the LH-LH-R axis may represent a valuable therapeutic strategy in EC.

## Materials and methods

### Production of the transgene construct

The mogpLuc2AhLH-R construct was assembled starting from the following fragments:

- Mogp-1: the *mogp-1* promoter was isolated by Polymerase Chain Reaction (PCR) from the pTKSV plasmid (kindly gifted by Prof. I. Miyoshi, Nagoya University, Japan^18^) using the Expand Long Template PCR system (Roche). The PCR protocol was as follows: denaturation at 94 °C for 2 min, 28 cycles at 94 °C for 10 s, 60 °C for 30 s, 68 °C for 2 min and a final extension step at 72 °C for 10 min.
- Luc: the luciferase gene was amplified by PCR from the pGL4.51[Luc2/CMV/Neo] vector (Promega), using the Phusion High-Fidelity DNA polymerase (Finnzymes, New England Biolabs), and applying the touchdown protocol, from 70 °C with a 2 °C decrease each 4 cycles, till 52 °C.
- 2A peptide: The 2A peptide sequence is inserted in frame between the cDNA of LH-R and the cDNA of Luciferase. It allows the co-translational cleavage of proteins (Luc and LH-R) produced by a unique cDNA. To generate a single cDNA with the luciferase/peptide 2A/LH-R/myc in frame we used PCR technique. A fragment of 400 bp was amplified with the primers Luc2Seq3F and 2ALhrREV (Supplementary Table S8), starting from 10 ng of the plasmid pBluescript SK + luciferase/peptide 2A. At the same time a fragment of 250 bp was amplified with the primers 2ALhrDIR and LhrIntREV (Supplementary Table S8) starting from 10 ng of the pCRblunt-LH-R vector. PCR conditions were the following: denaturation at 98 °C for 30 s min, 30 cycles at 98 °C for 10 s, 62 °C for 30 s, 72 °C for 2.30 min and a final extension cycle at 72 °C for 10 min. The DNAs amplified by these two reactions were combined and used for a subsequent PCR performed with the primers Luc2Seq3F and LHrIntREV (Supplementary Table S8). PCR conditions were the following: denaturation at 98 °C for 30 s min, 28 cycles at 98 °C for 10 s, 62 °C for 30 s, 72 °C for 30 s and a final extension cycle at 72 °C for 10 min. PCRs were performed using the Phusion High-Fidelity DNA polymerase (Finnzymes, New England Biolabs) according to the manufacturer’s protocol in 25 µl of final volume.
- LH-R: The hLH-R cDNA was amplified from RNA extracted from Hec1A cells (American Type Culture Collection, Manassas, VA), and retro-transcribed into cDNA, using the procedure described below, and the primers reported in Supplementary Table S8. For PCR amplification, the Phusion High-Fidelity DNA polymerase (Finnzymes, New England Biolabs) was used, and the touchdown protocol starting from 70 °C with a 2 °C decrease each 4 cycles, till 52 °C was applied, as above.

At the 3′ end of hLH-R cDNA, the sequence encoding the c-myc epitope was placed by PCR, inserting the myc sequence into the primer downstream to the hLH-R. The c-myc epitope was useful for easier identification of LH-R expressed by the transgene.

The list of the primers used for all the PCRs are listed in Supplementary Table S8.

The resulting construct (mogpLuc2AhLH-R) was assembled in the pBluescript SK (+) vector.

### Microinjection into oocytes and generation of transgenic mice

The 8000-bp mogpLuc2AhLH-R construct was excised with NotI restriction enzyme from the pBluescript SK(+) vector and microinjected into the male pronucleus of fertilized zygotes from FVB mice. Fertilized eggs were re-implanted into the oviduct of pseudo pregnant mice according to standard procedures^43^. All the procedures were accomplished at the LIGeMA laboratory of the University of Florence, Italy. This project was authorized by the Italian Ministry of Health with the authorization number 1241/2015. All the in vivo procedures have been performed according with ARRIVE (Animal Research: Reporting of In Vivo Experiments) guidelines.

### Screening of transgenic mice

Resulting puppies were screened by PCR on DNA extracted from tails through Chelex 100 (Chelex100 Resin, BIO RAD) and using the Platinum PCR Supermix (Invitrogen, Life Technologies). The primers we used are listed in Supplementary Table S8. PCR conditions were the following: denaturation at 94 °C for 2 min, 35 cycles at 94 °C for 30 s, 58 °C for 30 s, 72 °C for 30 s and a final extension cycle at 72 °C for 10 min. The two founders we identified, one male and one female, as well as their offspring were always screened as above and maintained in the heterozygous state in a FVB background.

### EC patients

126 nonconsecutive EC hysterectomy specimens collected from April 2013 to November 2015 at AOU Careggi, Florence, Italy, were enrolled in the study. Informed consent was obtained from each patient. All patients underwent surgery according to European Society of Gynecological Oncology (ESGO) guidelines. Patients were staged retrospectively according to the 2009 FIGO staging guidelines. The present study has been approved by the local Ethical Committee of AOU Careggi (Florence, Italy). All experiments on patients’ specimens were performed in accordance with relevant guidelines and regulations. All the patients given their written informed consent, in accordance with the Declaration of Helsinki.

### RNA extraction and reverse transcription (RT)

RNA was extracted from the ovaries and uteri of both wild type (WT) and TG mice using TRIzol reagent (Life Technologies, Thermo Fisher) according to the manufacturer’s protocol. Before the reverse transcription, RNA integrity was assessed with Bioanalyzer (Agilent 2100 Bioanalyzer). 1 µg of RNA was used for reverse transcription. Reverse transcriptase (SuperScript II Reverse Transcriptase, Invitrogen) and random primers were used according to manufacturer’s protocol. The reaction was performed with a first step at 25 °C for 10 min followed by 50 min at 42 °C and a final step at 70 °C for 15 min.

### Real-time quantitative PCR (RQ-PCR)

hLH-R mRNA expression was evaluated by real-time quantitative PCR (RQ-PCR) using 1 µl of cDNA. For the reaction SYBR green fluorescent dye (Power SYBR Green, Applied Biosystems) was used. For RQ-PCR on mice sample the expression levels of hLH-R were normalized to the levels of β-actin housekeeping gene. The primers pairs for hLH-R and β-actin were designed to span intron/exon boundaries, but are not able to distinguish human from mouse LH-R orthologs. The primer sequences for hLH-R are: 5′-TGCCTACCTCCCTGTCAAAG-3′ forward primer; 5′-TTGAGGAGGTTGTCAAAGG-3′ reverse primer. β-actin gene was used as housekeeping gene. The primer sequences for β-actin are: 5′-GGGGTGTTGAAGGTCTAAA-3′ forward primer; 5′-GATCTGGCACCACACCTTCT-3′ reverse primer^27^.

For RQ-PCR on human samples the expression levels of hLH-R and Kcnh2 were normalized to the levels of GAPDH housekeeping gene. For hLH-R primers we used the same described above. The sequences of Kcnh2 and GAPDH primers are the following: GAPDH sense 5′-GCTCTCGCTCCTCCTGTT-3′; GAPDH antisense 5′-ACGACCAAATCCGTTGACTC-3′; Kcnh2 sense 5′-ACGTCTCTCCCAACACCAAC-3′; Kcnh2 antisense 5′-GAGTACAGCCGCTGGATGAT-3′ (used at final concentration of 300 nM). Primers used for Kcnh2 are previously described in Pillozzi et al., 2018^44^.

PCR program was programmed with an incubation at 95 °C for 10 min, followed by 40 cycles of amplification: denaturation at 95 °C for 15 s and annealing–extension step at 60 °C for 1 min as described in Noci et al.^10^. The relative quantification of hLH-R expression levels was performed by the ΔΔCt method. Melting curve analysis were performed to exclude the amplification of a specific products or primer–dimer artefacts. Each reaction was performed in triplicate. Relative expression values of hLH-R were normalized for the expression value of Hec1A cells (low expression cell line).

### Gene expression analysis

Gene expression analysis was performed using Agilent one color Gene Expression slides with 44 k resolution (mouse GE 4X44K V2 microarray kit, Agilent Technologies). Purified RNA samples were prepared as described in Masselli et al.^45^ Raw data files obtained from the scanned slides were processed using Agilent Feature Extraction Software. Bioinformatic analysis were performed using the open source Rstudio software applying the Linear Models for Microarray Data (“LIMMA” package, Bioconductor) by Genomix4Life srl (Baronissi, Salerno, Italy). Each spot was first corrected for background intensity using the "normexp" approach of the background correct function. The between-array normalization and the quantile approach were performed according to the LIMMA package’s functions. To identify DE genes an average cut-off of twofold changes was considered. The identified DE genes were addressed as specific for the tumor when they were not found to be deregulated neither in the uteri from healthy TG mice or in the uteri from WT animals. Gene expression data discussed in this paper have been deposited in NCBI’s Gene Expression Omnibus and are accessible through GEO Series accession number GSE163488. The specimen characteristics of corresponding datasets in GSE163488 are reported in Supplementary Table S11.

### Enrichment and functional annotation analysis (FAA)

FAA of DE genes were analyzed using the Database for Annotation, Visualization and Integrated Discovery (DAVID) v6.8 Bioinformatic Database as reported in Magi et al.^46^. The analysis was based on Gene Ontology (GO) and Kyoto Encyclopedia of Genes and Genomes (KEGG) terms enrichment^47–49^ (KEGG copyright permission number: 210037).

### Cell transfection

Hec1A cells were transfected with the pBluescript SK(+) vector containing the mogpLuc2AhLH-R sequence by using lipofectamine 2000 (Invitrogen) according to the manufacturer's recommendations. The occurrence of the transfection was assessed by determining Luc activity through bioluminescence assay (see below).

### Bioluminescence assay

Two days after cell transfection we evaluated the light emitted by bioluminescent Luciferine. Luminescent data were obtained from cells either transfected with pBluescript SK(+) vector containing the mogpLuc2AhLH-R transgenic construct and transfected with the empty vector. Luciferine undergoes a luciferase-catalyzed oxidation resulting in an excited state that emits upon decaying to its ground state. The resulting sample light output was measured by using a current-measuring luminometer that read in arbitrary light units, usually referred to as “Relative Light Units” (RLU).

### Immunofluorescence (IF)

IF was performed on Hec1A cells previously transfected with the vector containing the mogpLuc2AhLH-R sequence. The anti c-myc antibody (4 μg/ml, Santa Cruz Biotechnology) was used as primary antibody, and the anti-mouse Alexa488 (1 μg/ml, Invitrogen, Thermo Fisher) as secondary antibody. Images were acquired with Nikon D-Eclipse C1 (Nikon) confocal microscope.

### Analysis of uterine morphometry

Mice were euthanized by inhalation of 100% CO_2_, the whole reproductive tract of female mice was excised and immediately fixed in 4% formaldehyde for 4 h, processed and embedded in paraffin following standard procedures. Lengthwise sections of 6 µm thick were prepared and put on positive-charged slides. Samples were stained with Hematoxylin and Eosin (H&E) following a standard protocol. The uterine radius (UR) was measured from the outer longitudinal smooth muscle layer (myometrium) to the apical surface of the luminal epithelium. The muscle layer was considered the inner circular layer. The luminal epithelial height (LEH) was measured from the basement membrane to the apical surface as described in Wood et al.^20^ All measurements were performed using a light microscope (Leica DMR, Germany) equipped with Leica DC Viewer and Leica Qwin software. The evaluation and measures were performed on the sample displaying the whole uterine cavity and at least three measurements per area were determined.

### Immunohistochemistry (IHC)

To block sample’s endogenous peroxidase, 1% H_2_O_2_ solution in phosphate-buffered saline was usen on dewaxed and dehydrated tissue slides. Antigen retrieval was performed by using different procedures: (1) dipping the samples in citrate buffer pH 6.0 and heated by microwave oven at 600 W for 12 min (for c-myc, Ki67, CK8 and α-sma staining) and (2) treating with proteinase K (5 μg/ml) in PBS at 37 °C for 5 min (for hERG1 staining). Samples were permeabilized with a 0.1% Triton X100 in UltraVBlock solution (LabVision) (for c-myc, Ki67 and α-sma staining) and incubated overnight at 4 °C with the following primary antibodies: anti-c-myc (monoclonal antibody, Santa Cruz Biotechnology, Santa Cruz, CA, 1:100), anti-cytokeratin-8 (Developmental Studies Hybridoma Bank, Iowa City, IA 1:100), anti-KI67 antigen (Dako, 1:50), anti α-sma (Dako, 1:100) and anti hERG1 (monoclonal antibody, MCK Therapeutics, 0.005 μg/μl). IHC was performed with commercially available kit (PicTure-Max polymer Detection kit, Invitrogen) according to manufacturer’s instruction. Hematoxylin was used for nuclear counterstaining. Immunohistochemistry slides were scored by three independent operators (T.L., J.I. and E.L.). For each protein, a specific scoring system was applied. α-sma expression was evaluated estimating the percentage of immunoreactive cells; samples were classified as positive when the percentage of labeled cells was equal to or greater than one. Ki67 staining was evaluated taking into account the percentage of labeled nuclei in three different areas: the luminal epithelium, stromal cells and the endometrial glandular cells. Stained sections were analyzed at a total magnification of 40 × field by field, from top left to bottom right. Previously published scoring system was applied for c-myc and hERG1^50^. Samples were scored combining the estimate of the staining intensity with the percentage of positive cells. Staining intensity was rated on a scale of 0–3, with 0 = negative; 1 = weak; 2 = moderate, and 3 = strong. The final scoring data were obtained by multiplying the percentage of labelled cells by the staining intensity values, obtaining a value between 0 and 300 for each sample. The c-myc and hERG1 scoring system was applied also to cytokeratin-8 for all samples.

### Statistical analysis

Statistical analyses were performed with OriginPro 8 (Origin Lab, Northampton, Massachusetts) on at least three independent experiments. The normality distribution of data the variance homogeneity were performed as previously described in Petroni et al.^51^

For comparisons between two groups, Student’s *t* test was used. In case of multiple comparisons, one-way ANOVA followed by Bonferroni’s post-hoc test was performed (as in^51^). All the data reported satisfy the assumptions of the tests. Test of normality distribution and variance homogeneity assumptions have been proper performed and used to choose the right test for compare groups.

For the human EC samples the statistical analysis the clinical-pathological parameters were categorized as fallows: histology = endometrioid vs non endometrioid; differentiation grade = low, medium, high; FIGO = I, II, III and IV; risk = low vs high; myometrial invasion = less than 50% versus greater than 50%. The associations between the values of expression LHR, Kcnh2 and clinical-pathological parameters were assessed applying the Spearman correlation test. *p* < 0.05 was considered statistically significant.

## Supplementary Information

Supplementary Information 1.

Supplementary Information 2.
