# Supplementary material for: Transgenic mice overexpressing the LH receptor in the female reproductive system spontaneously develop endometrial tumour masses
Source: Sci Rep. 2021 Apr 23;11:8847. doi: 10.1038/s41598-021-87492-5 (PMC8065064; doi:10.1038/s41598-021-87492-5)
Supplement: Supplementary file 2 — Supplementary Information 2. [file 41598_2021_87492_MOESM2_ESM.docx]

**Supplementary information**

**TRANSGENIC MICE OVEREXPRESSING THE LH RECEPTOR IN THE FEMALE REPRODUCTIVE SYSTEM SPONTANEOUSLY DEVELOP ENDOMETRIAL TUMOUR MASSES**

Tiziano Lottini ^1^, Jessica Iorio ^1^, Elena Lastraioli ^1^, Laura Carraresi ^2^, Claudia Duranti ^1^, Cesare Sala ^1^, Miriam Armenio ^1^, Ivo Noci ^3^ Serena Pillozzi ^1^ and Annarosa Arcangeli^1*^.

^1^ Department of Experimental and Clinical Medicine, University of Florence, Florence, Italy.

^2^ DI.V.A.L. Toscana srl, Sesto Fiorentino, Florence, Italy.

^3^ Department of Biochemical, Experimental and Clinical Science, University of Florence, Florence, Italy.

**Southern Blot analysis**

We evaluate the presence of the transgene in both TG-LHT-100 and TG-LHR-200 lines by Southern blot analysis. The genomic DNA was extracted from the tail with phenol/chloroform method. DNA was digested using EcoRI (unique site upstream intron/poliA) and separated on a 1% agarose gel, and afterwards blotted onto a nitrocellulose membrane. A DNA probe was generated on Luc2 DNA sequence and was radiolabelled using ^32^P-dCTP. Subsequently, the DNA blots were exposed to the hybridization probe and visualized by X-ray film by autoradiography.

**Supplementary Figure S1**: MogpLuc2AhLHR transgene detected by Southern blotting performed on tail genomic DNA of a F1 Tg-LHR-200 and Tg-LHR-100 mouse digested with EcoRI. Standard 1 and standard 2 are respectively 400 and 20 copies of MogpLuc2AhLHR construct. The transgene appeared to be integrated in a head-to-tail tandem array and the copies in TG-LHR-100 mouse was estimated slightly less than 20, while in TG-LHR-200 mouse the number of estimated copies was much lower.


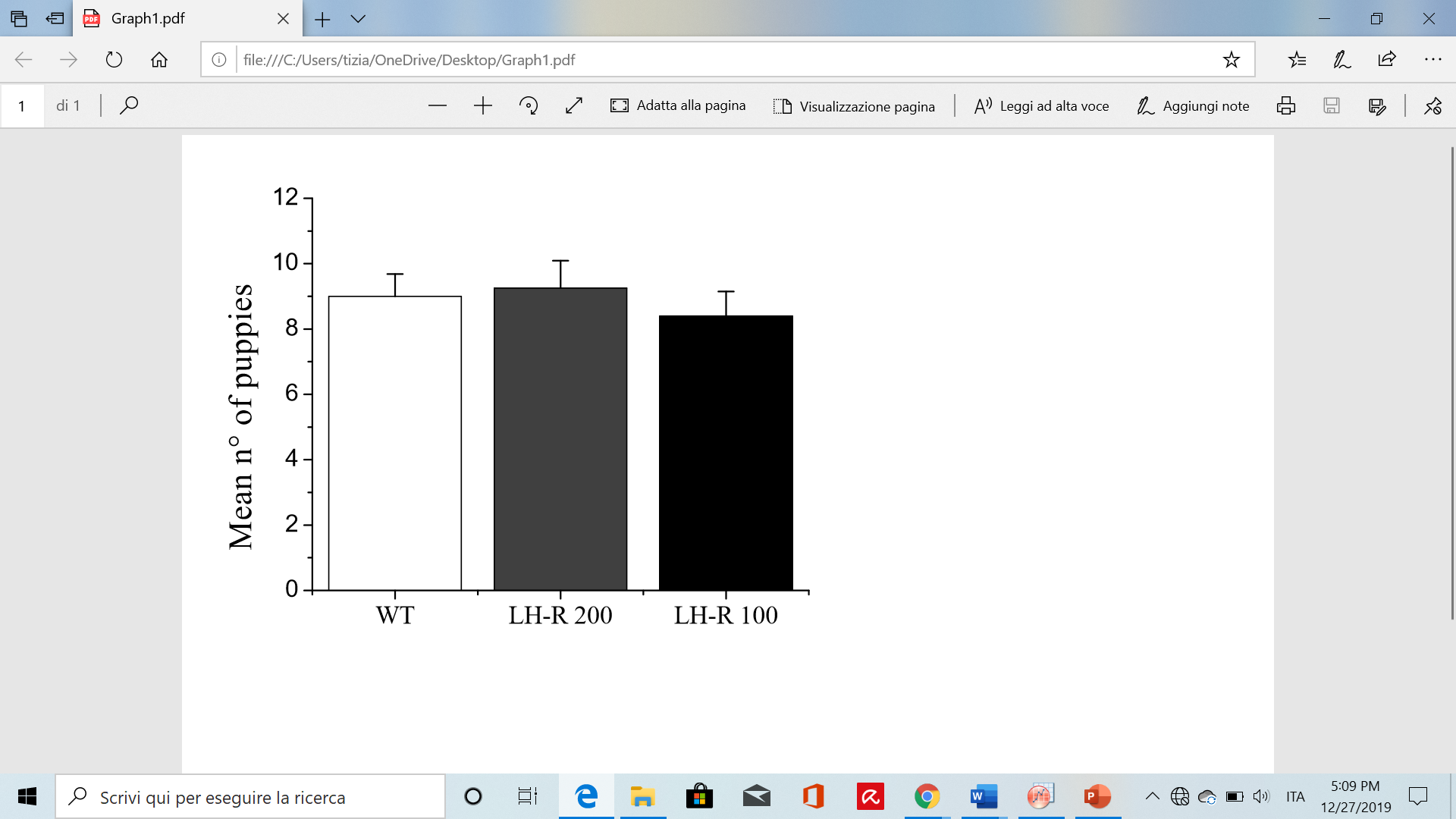


**Supplementary Figure S2:** Column plot representing the minimal differences between the mean number of puppies of FVB WT (9+0.6 white bar) mice and TG-LHR-frt-200 (in the graph abbreviated LH-R 200) (9.3+0.8 gray bar) and TG-LHR-frt-100 (in the graph abbreviated LH-R 100) (8.4+0.8 black bar) mice.


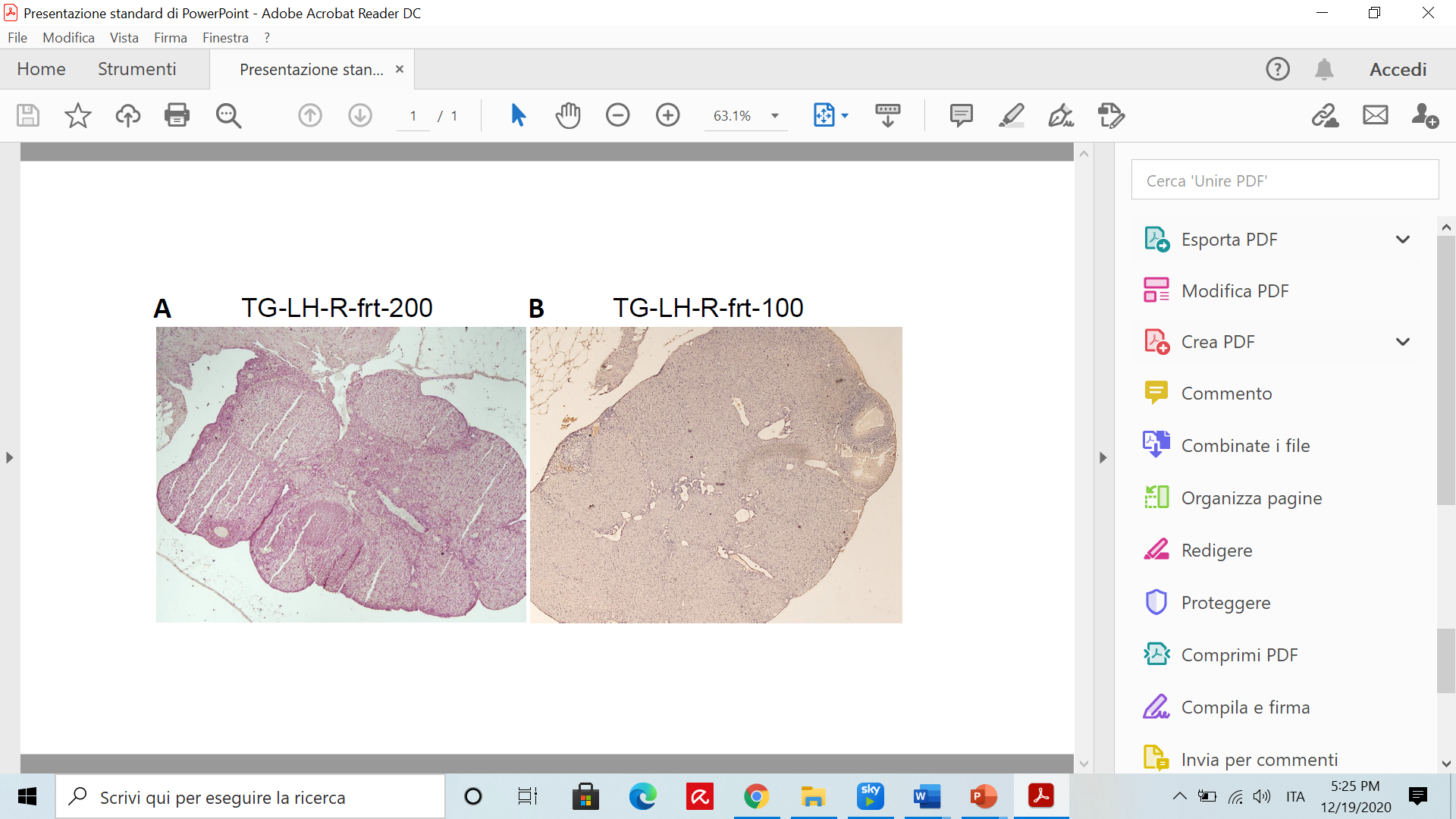


**Supplementary Figure S3:** H&E staining on representative ovary samples of TG-LHR-frt-200 mouse and TG-LHR-frt-100 mouse younger than 12 months of age.

|  | **Mean FC (2^-DDCT)** | | | | |
| --- | --- | --- | --- | --- | --- |
|  | **WT** | **TG-LH-R-frt-200** | **TG-LH-R-frt-200/WT** | **TG-LH-R-frt-100** | **TG-LH-R-frt-100/WT** |
| **Uterus** | 17.9 | 236 | *13.2* | 430 | *24* |
| **Ovary** | 14800 | 109981 | *7.4* | 229970 | *15.5* |
| **Liver** | 1.3 | 127 | *97.7* | 235 | *180.7* |
| **Spleen** | 13.5 | 269 | *19.9* | 460 | *34* |

**Supplementary Table S1:** Evaluation by RQ-PCR of the transcript level of LH-R mRNA in uterus, ovary, liver and spleen of WT and TG-LH-R-frt mice. For each mouse strain the values are reported as mean fold change (mean FC) and as ratio among the mean FC of the TG mouse and the WT (in italics).


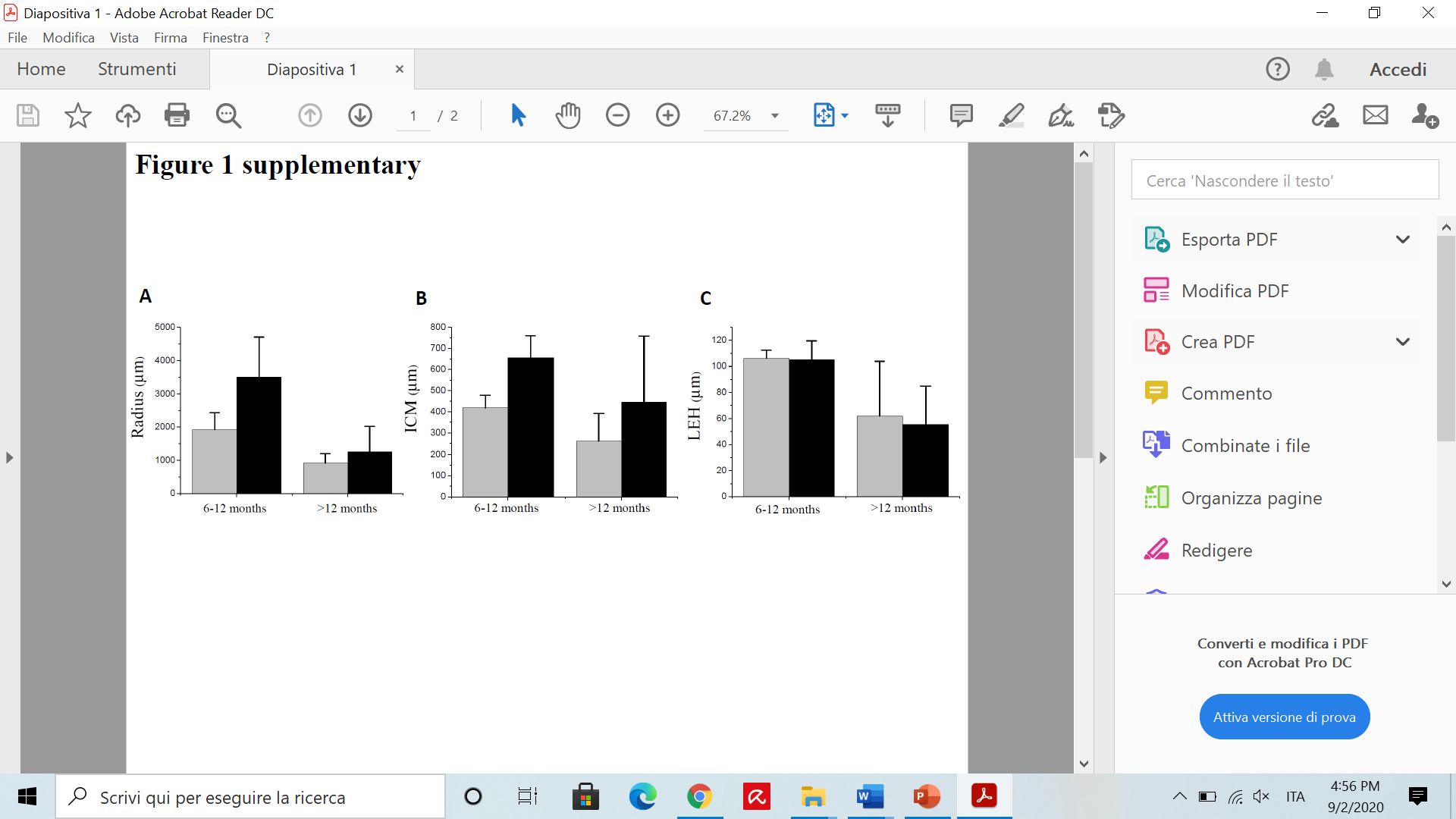


**Supplementary Figure S4** A, B, C: Mean of the Radius (B), ICM (C) and LEH (D) values of both wt (gray bars) and TG- LHR (black bars) mice. All the mice are divided into two groups: 3-12 months-old, and more than 12 months-old.


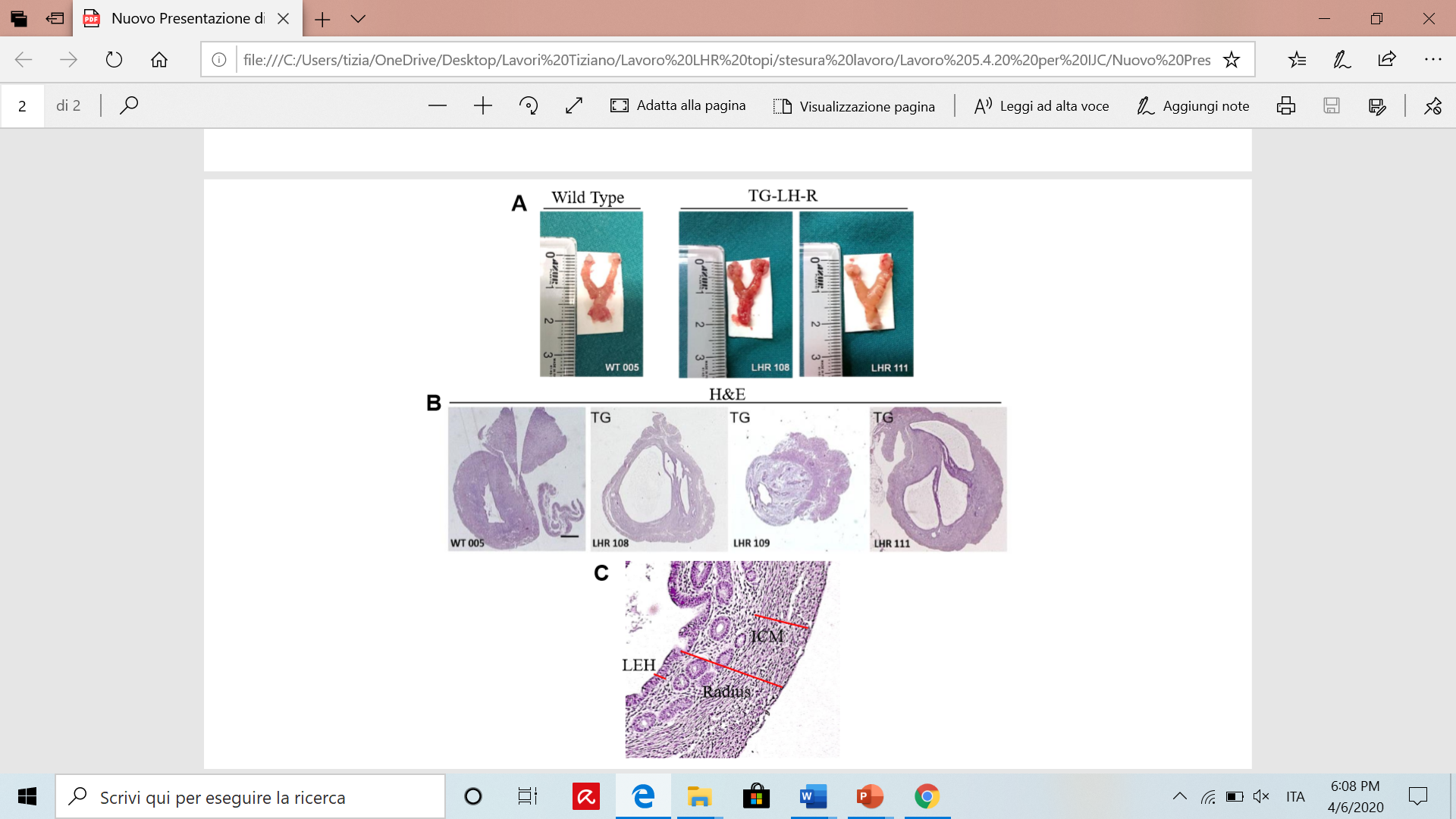


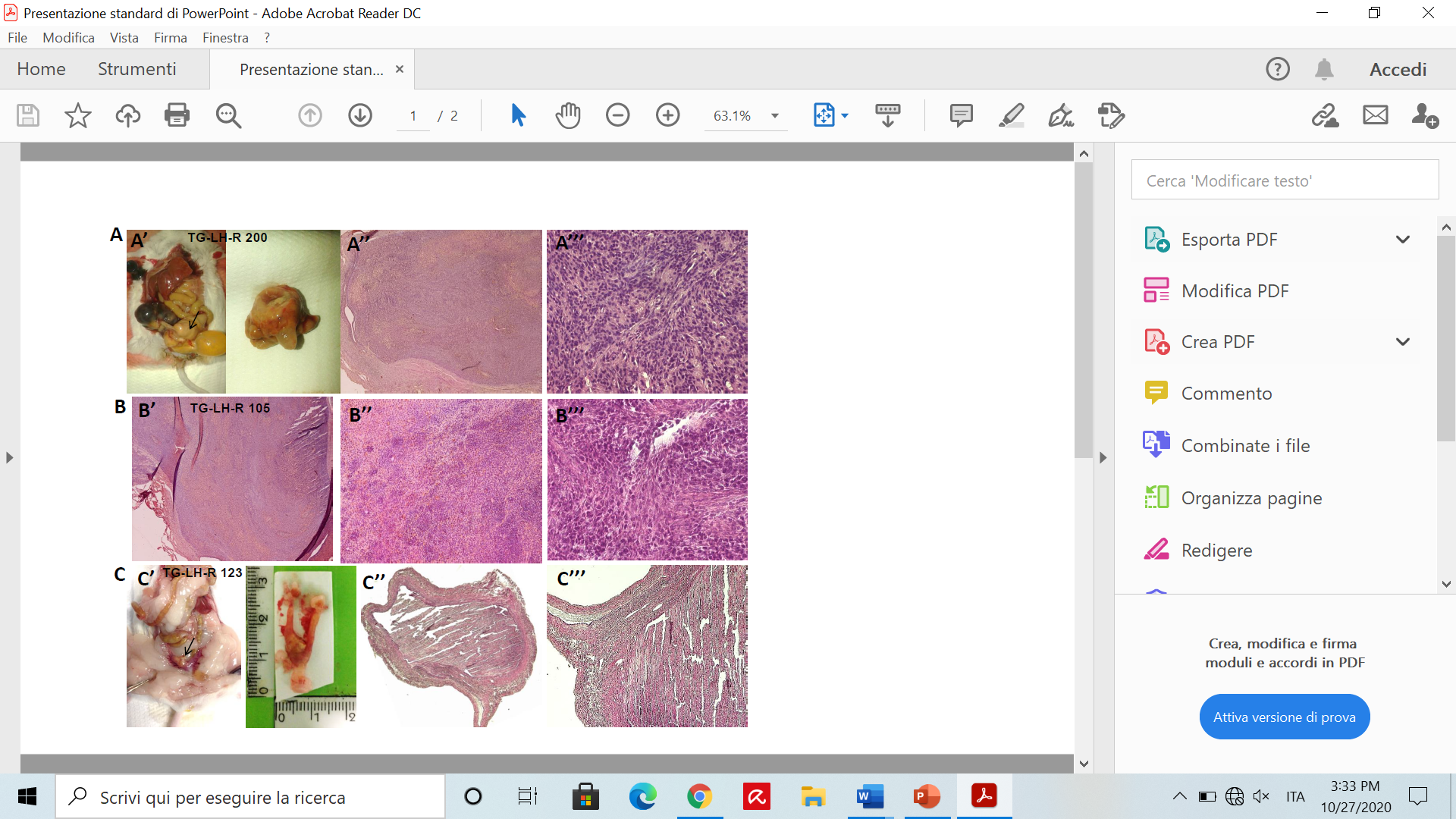
**Supplementary Figure S5. A:** Representative samples of explanted uteri. **B:** Hematoxylin/Eosin staining was performed on uterus of 9-months old WT and TG-LH-R mice. TG-LH-R-frt-108 and TG-LH-R-frt-111 showed an increased dimension of the uterus compared to a normal sized TG-LH-R-frt-109 mouse and WT 005 mouse. 1,6X magnification; 100 μm bar

**Supplementary Figure S6. A, B, C:** Gross view and low magnifications of the tumors observed in TG-LH-R-frt mice. **A:** View of the mass from TG-LH-R-frt-200 mouse at the moment of the explant **(A’)**. 4X magnification (**A’’**) and 40X magnification (**A’’’**) of H&E staining on the tumor mass. **B:** 4X magnification (**B’**), 10X magnification (**B’’**) and 40X magnification (**B’’’**) of H&E staining on the tumor mass observed on TG-LH-R-frt-105 mouse. **C:** View of the mass from TG-LH-R-frt-123 mouse at the moment of the explant (**C’**). 4X magnification (**C’’**) and 10X magnification (**C’’’**).

**Supplementary Table S2**

| **KEGG Pathway** | **Genes** |
| --- | --- |
| **Cell cycle** | MAD2 mitotic arrest deficient-like 1(Mad2l1) |
|  | Cell division cycle 20(Cdc20) |
|  | Cyclin A2(Ccna2) |
|  | Cyclin B1(Ccnb1) |
|  | Cyclin B2(Ccnb2) |
|  | Extra spindle pole bodies 1, separase(Espl1) |
|  | Growth arrest and DNA-damage-inducible 45 gamma(Gadd45g) |
| **FoxO signaling pathway** | Cyclin B1(Ccnb1) |
|  | Cyclin B2(Ccnb2) |
|  | Growth arrest and DNA-damage-inducible 45 gamma(Gadd45g) |
|  | Insulin-like growth factor 1(Igf1) |
|  | Polo-like kinase 2(Plk2) |
|  | Protein arginine N-methyltransferase 1(Prmt1) |
| **Focal adhesion** | RAS protein-specific guanine nucleotide-releasing factor 1(Rasgrf1) |
|  | Collagen, type V, alpha 1(Col5a1) |
|  | Collagen, type VI, alpha 3(Col6a3) |
|  | Insulin-like growth factor 1(Igf1) |
|  | Integrin beta 3(Itgb3) |
|  | p21 protein (Cdc42/Rac)-activated kinase 3(Pak3) |
| **Biosynthesis of antibiotics** | Adenosine monophosphate deaminase 3(Ampd3) |
|  | Cytochrome P450, family 51(Cyp51) |
|  | Farnesyl diphosphate synthetase(Fdps) |
|  | Isopentenyl-diphosphate delta isomerase(Idi1) |
|  | Methylsterol monoxygenase 1(Msmo1) |
|  | Squalene epoxidase(Sqle) |
| **p53 signaling pathway** | Cyclin B1(Ccnb1) |
|  | Cyclin B2(Ccnb2) |
|  | Growth arrest and DNA-damage-inducible 45 gamma(Gadd45g) |
|  | Insulin-like growth factor 1(Igf1) |
|  | Ribonucleotide reductase M2(Rrm2) |
| **Progesterone-mediated oocyte maturation** | MAD2 mitotic arrest deficient-like 1(Mad2l1) |
|  | Cyclin A2(Ccna2) |
|  | Cyclin B1(Ccnb1) |
|  | Cyclin B2(Ccnb2) |
|  | Insulin-like growth factor 1(Igf1) |

| **Oocyte meiosis** | MAD2 mitotic arrest deficient-like 1(Mad2l1) |
| --- | --- |
|  | Aurora kinase A(Aurka) |
|  | Cell division cycle 20(Cdc20) |
|  | Extra spindle pole bodies 1, separase(Espl1) |
|  | Insulin-like growth factor 1(Igf1) |
| **Protein digestion and absorption** | Collagen, type V, alpha 1(Col5a1) |
|  | Collagen, type VI, alpha 3(Col6a3) |
|  | Collagen, type XXII, alpha 1(Col22a1) |
|  | elastin(Eln) |
| **Steroid biosynthesis** | Cytochrome P450, family 51(Cyp51) |
|  | Methylsterol monoxygenase 1(Msmo1) |
|  | Squalene epoxidase(Sqle) |

**Supplementary Table S2:** Table showing enriched upregulated genes for each pathways, p<0.05.

**Supplementary Table S3**

| **KEGG_Pathway** | **Gene Name** |
| --- | --- |
| **PPAR signaling pathway** | CD36 antigen (Cd36)  acyl-CoA synthetase long-chain family member 1(Acsl1) adiponectin, C1Q and collagen domain containing (Adipoq) fatty acid binding protein 4, adipocyte (Fabp4)  fatty acid binding protein 5, epidermal (Fabp5) lipoprotein lipase (Lpl)  perilipin 1(Plin1)  peroxisome proliferator activated receptor gamma (Pparg) phosphoenolpyruvate carboxykinase 1, cytosolic (Pck1)  solute carrier family 27 (fatty acid transporter), member 1(Slc27a1) stearoyl-Coenzyme A desaturase 1(Scd1)  stearoyl-coenzyme A desaturase 3(Scd3) |
| **AMPK signaling pathway** | CD36 antigen (Cd36)  adiponectin, C1Q and collagen domain containing (Adipoq) glycogen synthase 2(Gys2)  insulin receptor substrate 2(Irs2) insulin receptor substrate 3(Irs3) lipase, hormone sensitive (Lipe)  peroxisome proliferator activated receptor gamma (Pparg) phosphoenolpyruvate carboxykinase 1, cytosolic (Pck1) protein phosphatase 2, regulatory subunit B', alpha (Ppp2r5a) stearoyl-Coenzyme A desaturase 1(Scd1)  stearoyl-coenzyme A desaturase 3(Scd3) |
| **Chemokine signaling pathway** | SHC (Src homology 2 domains containing) family, member 4(Shc4) chemokine (C-C motif) ligand 17(Ccl17)  chemokine (C-C motif) ligand 24(Ccl24) chemokine (C-C motif) ligand 4(Ccl4) chemokine (C-C motif) receptor 7(Ccr7) chemokine (C-X-C motif) ligand 1(Cxcl1) chemokine (C-X-C motif) ligand 10(Cxcl10) chemokine (C-X-C motif) ligand 5(Cxcl5) chemokine (C-X-C motif) receptor 2(Cxcr2) |
| **Regulation of lipolysis in adipocytes** | adenosine A1 receptor (Adora1) adrenergic receptor, beta 3(Adrb3)  fatty acid binding protein 4, adipocyte (Fabp4) insulin receptor substrate 2(Irs2) insulin receptor substrate 3(Irs3) lipase, hormone sensitive (Lipe)  perilipin 1(Plin1) |
| **Metabolism of xenobiotics by cytochrome P450** | aldehyde dehydrogenase family 1, subfamily A3(Aldh1a3) cytochrome P450, family 2, subfamily e, polypeptide 1(Cyp2e1) cytochrome P450, family 2, subfamily f, polypeptide 2(Cyp2f2) dihydrodiol dehydrogenase (dimeric)(Dhdh)  glutathione S-transferase, alpha 3(Gsta3) glutathione S-transferase, theta 1(Gstt1) hydroxysteroid 11-beta dehydrogenase 1(Hsd11b1) |
| **Insulin signaling pathway** | SHC (Src homology 2 domain containing) family, member 4(Shc4) glycogen synthase 2(Gys2)  insulin receptor substrate 2(Irs2) insulin receptor substrate 3(Irs3) lipase, hormone sensitive (Lipe)  phosphoenolpyruvate carboxykinase 1, cytosolic (Pck1) protein kinase, cAMP dependent regulatory, type II beta (Prkar2b) |
| **Adipocytokine signaling pathway** | CD36 antigen (Cd36)  acyl-CoA synthetase long-chain family member 1(Acsl1) adiponectin, C1Q and collagen domain containing (Adipoq) insulin receptor substrate 2(Irs2)  insulin receptor substrate 3(Irs3) phosphoenolpyruvate carboxykinase 1, cytosolic (Pck1) |
| **Chemical carcinogenesis** | aldehyde dehydrogenase family 1, subfamily A3(Aldh1a3) cytochrome P450, family 2, subfamily e, polypeptide 1(Cyp2e1) glutathione S-transferase, alpha 3(Gsta3)  glutathione S-transferase, theta 1(Gstt1) hydroxysteroid 11-beta dehydrogenase 1(Hsd11b1)  sulfotransferase family 1A, phenol-preferring, member 1(Sult1a1) |
| **Drug metabolism - cytochrome P450** | aldehyde dehydrogenase family 1, subfamily A3(Aldh1a3) cytochrome P450, family 2, subfamily e, polypeptide 1(Cyp2e1) flavin containing monooxygenase 5(Fmo5)  glutathione S-transferase, alpha 3(Gsta3) glutathione S-transferase, theta 1(Gstt1) |
| **Complement and coagulation cascades** | complement factor B(Cfb) complement factor D (adipsin)(Cfd)serine (or cysteine) peptidase inhibitor, clade A, member 1A(Serpina1a) serine (or cysteine) peptidase inhibitor, clade A, member 1C(Serpina1c) serine (or cysteine) peptidase inhibitor, clade A, member 1E(Serpina1e) |

**Supplementary Table S3:** Table showing enriched downregulated genes for each pathways, p<0.05.

| **Supplementary Table S4** |  |  |
| --- | --- | --- |
| **TG-hLH-R-frt vs WT uteri** | **Foxo1^d/d^ vs WT uteri** | **Wnt7a^cre/+^ mPgrA^LsL/+^ vs WT uteri** |
| **Upregulated KEGG Pathways** | **Upregulated Diseases or Functions Annotation** | **Altered biological functions** |
| Cell cycle | Cell survival | Lipid metabolism, molecular transport, small molecule biochemistry |
| FoxO signaling pathway | Vaso-occlusion | Organismal development |
| Focal adhesion | Cell viability | Reproductive system development and function |
| Biosynthesis of antibiotics | Overweight disorder | Cell-to-cell signaling and interaction, cellular growth, and proliferation |
| p53 signaling pathway | Obesity | Inflammatory response |
| Progesterone-mediated oocyte maturation | Occlusion of blood vessel | Connective tissue disorders, tissue morphology |
| Oocyte meiosis | Occlusion of artery |  |
| Protein digestion and absorption | Synthesis of fatty acid |  |
| Steroid biosynthesis | Ossification of bone |  |
|  | Immune response of cells |  |
| **Downregulated KEGG Pathways** | **Downregulated Diseases or Functions Annotation** |  |
| PPAR signaling pathway | Colorectal cancer |  |
| AMPK signaling pathway | Intestinal cancer |  |
| Chemokine signaling pathway | Intestinal tumor |  |
| Regulation of lipolysis in adipocytes | Adhesion of tumor cell lines |  |
| Metabolism of xenobiotics by  cytochrome P450 | Urogenital cancer |  |
| Insulin signaling pathway | Pelvic cancer |  |
| Adipocytokine signaling pathway | Chronic kidney disease |  |
| Chemical carcinogenesis | End stage renal disease |  |
| Drug metabolism - cytochrome P450 | Chronic renal failure |  |
| Complement and coagulation cascades | Proliferation of epithelial cell lines |  |
|  | Nonhematologic malignant neoplasm |  |
|  | Interaction of tumor cell lines |  |
|  | Digestive system cancer |  |
|  | Renal lesion |  |
|  | Malignant solid tumor |  |
|  | Solid tumor |  |
|  | Abdominal cancer |  |
|  | Seizures |  |
|  | Seizure disorder |  |
|  | Digestive organ tumor |  |
|  | Infection of mammalia |  |
|  | Parasitic Infection |  |
|  | Abdominal neoplasm |  |

**Supplementary Table S4:** Comparison between DE pathways and biological functions between the TG-LH-R-frt mouse model and FOXO1 uterine KO(Foxo1^d/d^) [23] and progesterone (Wnt7acre/+ mPgrALsL/+) overexpression models [24].

**Supplementary Table S5**

| **Category** | **Term** | **Count** | **Genes** | **FDR** |
| --- | --- | --- | --- | --- |
| GOTERM_BP_DIRECT | inflammatory response | 57 | S100A8, AIF1, LY86, S100A9, TLR2, TLR7, TGFB1, IL1B, ADAM8, PIK3CG, C5AR2, C5AR1, C4B, NCF1, CD40, CCR5, C3AR1, CCL2, CYSLTR1, CXCL9, CCL9, FPR1, PF4, NLRP1B, MAPKAPK2, FPR2, CCL4, CCL7, CCL6, CCL24, SLC11A1, TNFRSF1B, HRH1, NAIP2, MEFV, CHIL3, CYP26B1, PSTPIP1, NRROS, CSF1R, HCK, TLR13, AXL, ECM1, AIM2, CCL17, CCL12, CYBA, P2RX7, CYBB, CXCL13, STAB1, ALOX5,  CLEC7A, HDAC9, THEMIS2, CD14 | <0.001 |
| GOTERM_CC_DIRECT | membrane | 314 | ADCY7, S100A8, AIF1, PPP1R21, S100A9, CD52, CD53, ITSN1, TMEM141, BTK, CD48, HMHA1, S1PR1, CLEC4E, WWP1, OASL1, GNG2, CREB3L3, CLEC4D, ADAM8, HCAR2, SYK, PIK3CG, PTPRJ, C5AR2, F10, C5AR1, SCN2B, GATM, NCF2, NCF1, NCF4, PLD4, WNK1, TIMD4, GPR132, CD40, LPCAT2, PTPRO, SIRPA, CLEC4N, PLAUR, CD38, CD37, CCR5, CD33, CX3CR1, RYR1, PMP22, BIN1, TM6SF1, IL1R2, HFE, FCGRT, VMN1R48, CD72, CD74, SLC11A1, CD68, RAC2, FCER1G, SLC28A2, LFNG, TYROBP, SLAMF9, SLAMF6, TREX1, NCKAP1L, SLAMF8, ATP13A2, FCGR1, DAGLB, ABCG1, FCGR3, GNGT2, ADAP2, CD300A, COX6A2, PLAU, GPR84, PILRB2, PILRB1, MSR1, MPEG1, FERMT3, AP2S1, TRPV2, LY9, LTC4S, KCNK13, HVCN1, SCIMP, EFHD2, EDNRB, ST6GALNAC4, TNFRSF11A, DPEP2, PARVG, NFAM1, FCRLS, LRRC25, RAB11FIP5, DOK3, LRMP, MOSPD2, CLN8, ADAM15, SRGAP2, GPR183, GPR65, ABI3, HK2, FPR1, NLRP1B, FPR2, FKBP1B, APBB1IP, CLEC10A, LPXN, IL10RB, IL10RA, ALOX5AP, ABCD4, CERK, AMICA1, RASA4, CLEC2I, AATK, SOAT2, PLA2G15, TBXAS1, PLEK, SELL, ATP1A3, AXL, H2-AB1, TMPRSS5, P2RY12, SLC16A3, PIRB, MARCH1, RAB32, P2RY13, AP2A2, TFRC, PDE2A, FCGR2B, STAB1, CMTM7, RAB39, CD302, SLC15A3, CD14, GM11744, TSPAN4, TLR2, TNFSF18, DSE, TLR7, IL17RA, SLC7A7, MARCO, DAB2, MCOLN2, PQLC2, EBI3, BST1, H2-DMB1, TNFRSF17, HCST, IGSF6, OGFRL1, LAT2, ARRB2, TMEM106A, REM1, CD300LF, AKAP7, EMP3, CD300LB, CD300LD, FGD2, C3AR1, FGFR1, FXYD2, AB124611, LST1, ABCA9, CYSLTR1, ITLN1, HACD4, SLC38A6, SNX2, UNC93B1, MAP4K1, PAQR7, NINJ1, ITGB2, KMO, ABCA1, GBP2B, TRF, ITGAM, P2RY6, LAPTM5, FOLR2, NRROS, PLXND1, SLC35F6, CSF1R, ARHGDIB, MRC1, ADSSL1, RAB8B, NTPCR, TLR13, SLCO2B1, SREBF2, P2RX4, LYVE1, P2RX7, CORO1A, GPR34, ST8SIA4, H2-EB1, TRAF3IP3, MILR1, SLC13A3, CACNA1F, CPNE2, PIP4K2A, MERTK, IL3RA, SLC45A4, AI467606, SGPP1, TNFSFM13, HEXA, PREX1, IL21R, ARHGAP15, FES, SYNGR1, CBFA2T3, SDC3, MCTP2, HPSE, EVI2A, HMOX1, NECAP2, CSF2RB, AHNAK, SLC43A2, PILRA, LAIR1, ADGRE1, PIGZ, MS4A6C, MPP1, MS4A6D, ADGRE5, BTNL2, MCEMP1, CTSS, ALDH3B1, CD84, MAN2A2, STOM, CD83, SIGLEC1, ADRB2, CD86, DHRS3, CD80, H2-AA, CTSC, TREM2, LCP1, FCNA, APH1B, CYTH4, GM5431, COMT, NR3C1, TMEM37, HRH1, TNFRSF1B, RASGRP2, TSPAN32, CYP26B1, PSTPIP1, SLC25A44, SH2B2, IL2RG, PIK3R6, INPP5D, MS4A6B, SELPLG, SNX20, PSTPIP2, PTPRE, HCK, RGS14, ANXA3, GSG1, IL6RA, CYBA, CYBB,  ITPRIPL2, EPOR, TMEM86A, ALOX5, IFI203, H2-DMA | <0.001 |
| GOTERM_BP_DIRECT | positive regulation of ERK1 and ERK2  cascade | 20 | GPR183, C5AR2, CCL2, C5AR1, CCL9, TLR2, CCL4, SCIMP, CCL7, CD74, TGFB1, CCL6, CCL17, CCL24, CCL12, ARRB2, IL1B, EPOR, TREM2, CSF1R | 0.001 |
| GOTERM_BP_DIRECT | chemokine- mediated signaling pathway | 11 | CCL24, CCL12, CCL2, CXCL13, CCL9, CXCL9, PF4, CCL4, CCL7, CCL6, CCL17 | 0.003 |
| GOTERM_BP_DIRECT | cellular response to interferon- gamma | 12 | CCL24, MRC1, CCL12, CCL2, AIF1, CCL9, H2-AB1, GBP2B, CCL4, CCL7, CCL6, CCL17 | 0.003 |
| GOTERM_BP_DIRECT | signal transduction | 62 | GPR84, TLR2, ARHGAP15, TLR7, RGL1, ARHGAP4, CD48, EDNRB, HMHA1, S1PR1, STARD8, GNG2, HCAR2, ARHGAP9, PILRA, IRS2, ADGRE1, C5AR2, C5AR1, ADGRE5, TNFRSF17, GPR132, NFAM1, ARHGAP25, ARHGAP30, CD83, ADRB2, ARRB2, CCR5, CX3CR1, REM1, CAMK1, INPP4A, SRGAP2, GPR183, C3AR1, CYSLTR1, GPR65, FPR1, VMN1R48, FPR2, APBB1IP, CD74, PLCL2, P2RY6, HRH1, FCER1G, SH2B2, PLXND1, RASA4, FYB, TLR13, ECM1, RGS14, CCL17, P2RY12,  P2RY13, P2RX4, GNGT2, GPR34, PDE2A, EPOR | 0.009 |
| GOTERM_BP_DIRECT | positive regulation of GTPase activity | 16 | CCL2, PREX1, CCL9, CD40, CCL4, VAV1, CCL7, CCL6, CCL17, CCL24, RGS10, CCL12, S1PR1, RASGRP2, BIN1, SRGAP2 | 0.017 |
| GOTERM_MF_DIRECT | cytokine receptor activity | 9 | IL21R, CX3CR1, CSF2RB, EPOR, IL2RG, CD74, IL3RA, EBI3, IL6RA | 0.034 |
| GOTERM_BP_DIRECT | positive regulation  f angiogenesis | 14 | CCL24, C3AR1, CYBB, C5AR1, CYSLTR1, HMOX1, C6, CX3CR1, SERPINE1, IL1B, ITGB2, PIK3R6, ECM1, ANXA3 | 0.041 |
| **Supplementary Table S5.** Upregulated genes, identified by FAA, significantly associated to terms with a FDR p value < 0.05. | | | | |

| **Supplementary Table S6** | | | | |
| --- | --- | --- | --- | --- |
| **Category** | **Term** | **Count** | **Genes** | **FDR** |
| GOTERM_CC_DIRECT | Extracellular exosome | 209 | LDHB, RARRES2, ATP1B1, IL6ST, LTBP3, EFNA1, LTBP4, AQP1, ATP2B2, ACTG2, OGN, HTRA1, VNN1, RAB25, RAPGEF3, CFI, PI16, SH3GL3, F11R, DAB2IP, PTPRF, PCBD1, CRYAB, TMEM132A, SERPING1, PIK3IP1, KRT19, NAPEPLD, KRT18, SERPINF1, F3, ST14, ABAT, RAB15, WFDC2, GCNT3, ENPP3, PTH1R, SERPINH1, EPHB4, SEMA5A, SERINC2, PFN2, LAMB2, FAM213A, SERPINA1A, SERPINA1D, UPK1A, SERPINA1C, AGRN, LAMB1, COL18A1, S100A16, S100A11, PTPN13, SERPINA1E, ACPP, NCKAP1, LAMA2, CBLC, GGT6, CD55, SYNE2, LAMA5, HEBP2, CDC42BPA**, TGFBR3,** FCGBP, SYTL1, PXDN, 2310030G06RIK, GREB1, ANPEP, VPS37D, SEMA3C, SEMA3B, TRPV6, NT5E, DPP4, VWA2, ACTA2, ICAM2, LRRC26, CLIC5, GNAS, SLC27A2, SHROOM2, EPDR1, C3, JCHAIN, CCL28, ITM2A, EZR, GPM6A, CRISPLD2, FAT1, CKMT1, PKD2, GALNT16, MYO5B, CAR2, TMPRSS2, HSPG2, NID1, QPCT, PROM1, PLSCR1, FBLN1, TOM1L1, CDH16, ATP6V0A4, LRP2, TSPAN1, SLC44A2, SLC44A4, TSPAN8, CXCL12, GSTM5, GSTM7, MMRN2, PACSIN3, 5330417C22RIK, SLC2A4, ROBO4, SPON2, NCALD, SPARCL1, MGP, MFGE8, NCAM1, CDKL1, CPE, FNBP1L, HSPB1, DSP, FAM171A1, FXYD3, CLU, ITGB4, ITGB3, TIMP3, PPL, NDRG2, ENTPD2, CPNE5, EFEMP1, MYO1D, PDZK1IP1, LCN2, TST, NEDD4, PLEKHA7, MYH11, MYH14, ANTXR1, MYLK, RHOJ, AEBP1, CLDN3, CLDN5, ANO1, IGFBP6, SORL1, LSR, DDR2, DSTN, CTTN, DES, GSN, LTF, LBP, SLC1A1, PADI2, SPINT1, PADI1, SMO, DDR1, EVPL, IGF2R, CNTN1, PPM1L, PLLP, PRNP, PRKCZ, GPRC5C, LUM, CD248, **CDH2,** CPZ, PRSS8, ALDH1A1, ISLR, KRT7, KRT8, TGM2, BDH2, GSTO1, SLC39A4, DPT,  COBLL1, GSTA3, TNXB, TMC4, HID1, XPNPEP2, IRF6, DSC2, C1S1, C1S2 | <0,001 |
| GOTERM_BP_DIRECT | epithelial cell differentiation | 14 | F11R, ELF3, CNN3, TBX1, EHF, WT1, TST, TAGLN, GATA6, UPK1A, CES1D, BDH2, TGFB1I1, BMP7 | 0.002 |
| GOTERM_CC_DIRECT | apical plasma membrane | 32 | PRKCZ, ATP1B1, SHROOM2, OCLN, ERBB2, PTH1R, ANO1, **CDH2,** ITGB3, AQP1, DDR2, ATP2B2, EZR, MARVELD2, UPK1A, SLC4A7, SLC39A4, SLC1A1, DPP4, PLET1, MAL, KCNK1, FZD6, PROM1, TJP1, CD55, CD34, S100G, TJP3,  AaTP6V0A4, LRP2, EMP2 | 0.006 |
| GOTERM_BP_DIRECT | positive regulation of cell migration | 24 | BMP4, COL18A1, PLET1, GCNT2, BCAR1, SPHK1, CX3CL1, ITGB3, AQP1, CXCL12, IRS1, IGF1R, SYNE2, SEMA3F, ROR2, SEMA3C, SEMA3B, JAK2, **LAMC2,** LAMB1, FAM83H, TRIP6, GRB7, MYLK | 0.008 |
| GOTERM_BP_DIRECT | positive regulation of transcription from RNA polymerase II promoter | 69 | FOXA2, ELF3, GLI3, GLI1, PGR, GATA2, OSR2, GATA6, SDPR, PAX8, CREB3L1, SOX18, YAP1, SOX17, RAPGEF3, MYB, IHH, AR, SSBP3, DAB2IP, ESR1, LDB2, SIX5, SIX4, HMGA2, GRHL2, MYCN, LPIN3, HOXD9, NCK2, SMO, **MSX1**, WNT5A, ABLIM3, LUM, GLIS2, EHF, DCN, MEIS1, WT1, PLAGL1, MEIS2, HAND2, HOXA10, PKD2, NFATC4, AGRN, KLF5, BMP4, EPAS1, TEAD1, TBX1,  TEAD2, WWTR1, PLAC8, CAPRIN2, PLSCR1, EBF4, SFRP2, CDON, HOXB5, SALL1, ID4, PBX1, JAK2, EAF2, BMP7, BMPR1A, NFIB | 0.009 |
| GOTERM_BP_DIRECT | bicellular tight junction assembly | 9 | RAMP2, PARD3, OCLN, CLDN3, CGN, MARVELD2, MARVELD3, PTPN13, GRHL2 | 0.010 |
| GOTERM_BP_DIRECT | positive regulation of epithelial cell proliferation | 14 | WNT5A, BMP4, DAB2IP, ERBB2, ESR1, TBX1, GAS1, SMO, OSR2, HTRA1, ESRP1, ESRP2, IHH, BMPR1A | 0.012 |
| GOTERM_CC_DIRECT | focal adhesion | 35 | MPZL1, LIMS2, PDLIM7, CNN3, BCAR1, WASF1, FERMT2, AKAP12, PDLIM1, CDH2, ITGB3, DDR2, CTTN, EZR, SORBS1, GSN, FAT1, TGM2, CNN1, AFAP1,  DPP4, FLRT1, EFNB2, HSPG2, SYNPO2, FLNC, NEXN, NCKAP1, PGM5, SYNE2, IGF2R, HSPB1, TGFB1I1, TRIP6, GRB7 | 0.012 |
| GOTERM_MF_DIRECT | calcium ion binding | 51 | EPDR1, LTBP3, ENPP2, LTBP4, CD248, PAMR1, **CDH2,** MMRN1, KCNIP3, MYL9, SMOC2, ATP2B2, GSN, SLC24A3, SMOC1, FAT1, PKD2, TGM2, AGRN, PLS3, THBS3, VWA2, IHH, MATN2, S100A16, EGFL7, SCUBE2, SPARCL1, NCALD, EFEMP1, HSPG2, S100A11, MGP, PADI2, NID1, CELSR1, PCDH17, PADI1, ITPR1, PLSCR1, CBLC, FBLN1, CDH16, S100G, DSC2, MCC, LRP2, C1S1, SYTL1, C1S2,  VLDLR | 0.022 |
| GOTERM_BP_DIRECT | positive regulation of angiogenesis | 17 | WNT5A, RAMP2, C3, SPHK1, CX3CL1, ITGB3, AQP1, SEMA5A, GATA2, PTGIS, GATA6, CD34, SFRP2, F3, HSPB1, CMA1, RAPGEF3 | 0.035 |
| GOTERM_CC_DIRECT | cytoskeleton | 71 | **CTNNAL1**, LZTS2, PDLIM7, MYBPC1, WASF1, FERMT2, PDLIM1, RASSF7, ACTG2, CTTN, IFT122, DES, PBXIP1, GSN, FAM83H, JAKMIP1, DHCR24, DYNC1I1, ACTA2, FILIP1, MICAL3, CAMSAP3, PLEKHH1, FLNC, NEXN, LRRC26, FARP1, RSPH9, TACC2, EML2, EVC2, SMTN, PGM5, EML1, EVPL, FNBP1L, SIPA1L1, CLIC5, HSPB1, DSP, TGFB1I1, KAZN, COBL, PARD3, SHROOM2, B9D1, AKAP12, IGF2BP2, TPM2, TPM1, PFN2, EZR, SORBS1, PPL,  RNF128, GPSM2, TCTN2, CNN1, PPP2R2B, AFAP1, CDC42EP5, EPPK1, PTPN13, SYNE2, PLEKHA7, NSMF, SYNM, JAK2, MAP6, TRIP6, MYLK | 0.049 |
| **Supplementary Table S6.** Downregulated genes, identified by FAA, significantly associated to terms with a FDR p value < 0.05. | | | | |

| **Supplementary Table S7:** Pathological findings in TG-hLH-R-frt mice | | | | |
| --- | --- | --- | --- | --- |
| Age (months) | Genotype | No. of mice (total) | Uterine histology | No. of mice with indicated histology |
| 3-12 | TG-hLH-R-frt-100 | 6 | Hyperplasia* | 2 |
|  |  |  | Trans-differentiation** | 2(out of 2 analysed) |
|  |  |  | Endometrial carcinoma | 0 |
|  | TG-hLH-R-frt-200 | 6 | Hyperplasia* | 0 |
|  |  |  | Trans-differentiation** | 2(out of 2 analysed) |
|  |  |  | Endometrial carcinoma | 0 |
| 12-17 | TG-hLH-R-frt-100 | 4 | Hyperplasia* | 0 |
|  |  |  | Trans-differentiation** | 1(out of 1 analysed) |
|  |  |  | Endometrial carcinoma | 0 |
|  | TG-hLH-R-frt-200 | 5 | Hyperplasia* | 0 |
|  |  |  | Trans-differentiation** | 1(out of 1 analysed) |
|  |  |  | Endometrial carcinoma | 0 |
| > 17 | TG-hLH-R-frt-100 | 4 | Hyperplasia* | 0 |
|  |  |  | Trans-differentiation** | 1(out of 1 analysed) |
|  |  |  | Endometrial carcinoma | 2 |
|  | TG-hLH-R-frt-200 | 5 | Hyperplasia* | 0 |
|  |  |  | Trans-differentiation** | 1(out of 1 analysed) |
|  |  |  | Endometrial carcinoma | 1 |
| * sample Ki67+ and CK8+ | | | | |
| ** α-sma+ staining in glandular epithelial cells | | | | |

**Supplementary Table S7:** Table summarizing the pathological findings in TG-hLH-R-frt mice.

| **Supplementary Table S8** | |
| --- | --- |
| **Primers used for amplifications** | **Primer sequence** |
| *Spe1Luc2up (forward)* | c**actagt**gccacc**atg**gaagatgccaaaaac |
| *EcoR12ALuc2dn* (2A underlined) (reverse) | ccttaagtgggccaggattctcctcgacgtcaccgcatgttagcagacttcctctgccctctccactgcc*cacggcgatcttgccgcccttc* |
| *EcoVLHrup (forward)* | gggatatcatgaagcagcggttctcggcgctg |
| *BamH1LHrdn (reverse)* | cccctaggttaacactctgtgtagcgagtcttgtc |
| *SalIMycLHrdn* (stop codon is bold, Myc is underlined) | gcgcagctg**tca**cagatcctcttctgagatgagtttttgttcacactctgtgtagcgagtcttg |
| *2ALHrDir* (underlined 2A,  LH-R bold) | gtgacgtcgaggagaatcctggccca**atgaagcagcggttctcggcg** |
| *2ALHrRev* (Underlined 2A, LH-R bold) | **cgccgagaaccgctgcttcat**tgggccaggattctcctcgacgtcac |
| *LhrIntRev* | ctgagagatttcaatttttatgacc |
| *Notmogp1upnew* | cgcggccgccaagttgggctggtcactgttac |
| *Mogp1Spedn* | gactagtgccctggtagctctggtga |
| **Primers used for the screening of TG-LH-R mice** |  |
| forward | GGCTGAAGAGCCTGATCAAATACA |
| reverse | CGCATGTTAGCAGACTTCCTCT |
| **Primers used for confirming the DE genes from microarray data** |  |
| Ccl24_F | TATCTCCAGGATCTCTTTTCTG |
| Ccl24_R | ATGGTCACAGAATCTATGGG |
| Cdc20_F | AAAAAGGAGCATCAGAAAGC |
| Cdc20_R | GGATGTATCTGCAAGCTCTTC |
| Cccna2_F | AGCAATGTTTTTGGGAGAAC |
| Cccna2_R | AGGGTATATCCAGTCTGTTG |
| Ccnb2_F | GCCAAGGAAAATGGAATTTG |
| Ccnb2_R | TACGGTTGTCATTGACTTTC |
| Igf1_F | GACAAACAAGAAAACGAAGC |
| Igf1_R | ATTTGGTAGGTGTTTCGATG |
| Gsta3_F | TTGAAAAGGTGTTGAAGAGC |
| Gsta3_R | AAGAAACTTCTTCACTGTGG |
| Ccnb1_F | ACATGACTGTCAAGAACAAG |
| Ccnb1_R | CAGATGTAGCAGTCTATTGG |
| Cxcl1_F | AAAGATGCTAAAAGGTGTCC |
| Cxcl1_R | GTATAGTGTTGTCAGAAGCC |

**Supplementary Table S8**: the primers used in the production of the mogpLuc2AhLH-R construct are reported in the table.

| **Supplementary Table S9** | |
| --- | --- |
| **Common DE genes between tumor mass of TG-hLH-R-frt-200 and data analysis in O’Mara et al. 2016** | |
| **Upregulated genes** | **Description** |
| Ryr1 | ryanodine receptor 1, skeletal muscle |
| **Downregulated genes** | **Description** |
| Ehf | ets homologous factor |
| Sytl1 | synaptotagmin-like 1 |
| Gcnt3 | glucosaminyl (N-acetyl) transferase 3, mucin type |
| Tmc4 | transmembrane channel-like gene family 4 |
| Tst | thiosulfate sulfurtransferase, mitochondrial |
| Sox17 | SRY (sex determining region Y)-box 17 (Sox17), transcript variant 1 |
| Tmprss2 | transmembrane protease, serine 2 |
| Tjp3 | tight junction protein 3, transcript variant 3 |
| Marveld2 | MARVEL (membrane-associating) domain containing 2, transcript variant 1 |
| Esr1 | estrogen receptor 1 (alpha), transcript variant 1 |
| **Common DE genes between tumor mass of TG-hLH-R-frt-200 and data analysis in Liu et al. 2018** | |
| **Downregulated genes** | **Description** |
| Hand2 | heart and neural crest derivatives expressed transcript 2 |
| Tgfbr3 | musculus transforming growth factor, beta receptor III |

**Supplementary Table S9:** List of common DE genes between the tumor mass of TG-hLH-R-frt-200 mouse confirmed by comparing it with datasets analyzed in O’Mara 2016 and the dataset analyzed in Liu 2018 were chosen for the comparison.

| **Supplementary Table S10** | | | | | | | | | | |
| --- | --- | --- | --- | --- | --- | --- | --- | --- | --- | --- |
| **Sample code** | **KCNH2**  **fold** | **LHR**  **fold** | **FIGO**  **stage^I^** | **Age at diagnosis** | **Histotype^II^** | **Risk^III^** | **Grading ^IV^** | **MI**  **>50%^V^** | **Menopause^VI^** | **BMI** |
| **Fi-1** | 176.48 | 1109644,92 | 1 | 74 | 1 | 0 | 2 | 0 | 1 | 41 |
| **FI-2** | 4.08 | 5722972,86 | 1 | 61 | 1 | 1 | 3 | 1 | 1 | 29 |
| **FI-3** | 12.47 | 2,26 | 1 | 79 | 1 | 1 | 2 | 1 | 1 | 24 |
| **FI-4** | 260.78 | 2,70 | 1 | 56 | 1 | 0 | 2 | 0 | 0 | 22 |
| **FI-5** | 5.72 | 15,35 | 1 | 73 | 1 | 1 | 2 | 1 | 1 | 29 |
| **FI-6** | 95.56 | 2,14 | 1 | 74 | 1 | 1 | 2 | 1 | 1 | 25 |
| **FI-8** | 107.88 | 3603,04 | 1 | 64 | 1 | 0 | 1 | 0 | 1 | 29 |
| **FI-9** | 6.11 | 134,83 | 1 | 54 | 1 | 1 | 2 | 1 | 0 | 25 |
| **FI-10** | 26.66 | 25,81 | 2 | 79 | 0 | 1 | 3 | 1 | 1 | 29 |
| **FI-12** | 2.38 | 47,50 | 1 | 54 | 1 | 0 | 1 | 0 | 1 | 25 |
| **FI-13** | 6746.86 | 858,10 | 1 | 70 | 1 | 0 | 2 | 0 | 1 | 31 |
| **FI-14** | 766.25 | 350,92 | 1 | 63 | 1 | 0 | 1 | 0 | 1 | 35 |
| **FI-15** | 421.19 | 144,51 | 1 | 53 | 1 | 0 | 2 | 0 | 1 | 23 |
| **FI-16** | 2.51 | 3385,14 | 3 | 65 | 0 | 1 | 3 | 1 | 1 | 29 |
| **FI-17** | 392.08 | 75,06 | 1 | 64 | 1 | 0 | 1 | 0 | 1 | 38 |
| **FI-18** | 6532.11 | 23657,34 | 1 | 65 | 1 | 0 | 2 | 0 | 1 | 37 |
| **FI-20** | 8.21 | 50,74 | 1 | 71 | 1 | 0 | 2 | 0 | 1 | 29 |
| **FI-21** | 0.67 | 3,49 | 1 | 65 | 1 | 0 | 2 | 0 | 1 | 34 |
| **FI-22** | 346.09 | 99,73 | 1 | 68 | 1 | 1 | 2 | 1 | 1 | 35 |
| **FI-23** | 11268.44 | 55,72 | 1 | 67 | 1 | 1 | 2 | 1 | 1 | 32 |
| **FI-24** | 407.31 | 15,35 | 1 | 67 | 1 | 0 | 2 | 0 | 1 | 34 |
| **FI-25** | 59.03 | 0,94 | 1 | 55 | 1 | 0 | 2 | 0 | 1 | 32 |
| **FI-26** | 165.04 | 0,26 | 1 | 76 | 1 | 0 | 1 | 0 | 1 | 24 |
| **FI-27** | 8.28 | 41,21 | 1 | 75 | 0 | 1 | 3 | 1 | 1 | 25 |
| **FI-28** | 30.48 | 1734,13 | 1 | 56 | 1 | 1 | 2 | 1 | 1 | 28 |
| **FI-29** | 14.16 | 4482,23 | 2 | 73 | 1 | 0 | 2 | 0 | 1 | 37 |
| **FI-31** | 2.81 | 44,17 | 3 | 71 | 0 | 1 | 2 | 1 | 1 | 37 |
| **FI-32** | 113.77 | 8220,44 | 1 | 70 | 1 | 0 | 1 | 0 | 1 | 24 |
| **FI-33** | 16.41 | 2,62 | 1 | 73 | 0 | 1 | 3 | 1 | 1 | 47 |
| **FI-34** | 377.85 | 1128,35 | 1 | 61 | 1 | 0 | 2 | 0 | 1 | 22 |
| **FI-35** | 4034.95 | 373,51 | 1 | 70 | 1 | 0 | 2 | 0 | 1 | 24 |
| **FI-36** | 184.82 | 491,14 | 1 | 65 | 1 | 0 | 2 | 0 | 1 | 37 |
| **FI-37** | 37.19 | 5,86 | 1 | 72 | 1 | 1 | 2 | 1 | 1 | 23 |
| **FI-38** | 9.27 | 9,75 | 1 | 59 | 1 | 0 | 2 | 0 | 1 | 41 |
| **FI-39** | 4.87 | 85,33 | 1 | 59 | 1 | 0 | 2 | 0 | 1 | 35 |
| **FI-40** | 61.39 | 2,11 | 2 | 51 | 1 | 1 | 3 | 1 | 0 | 30 |
| **FI-41** | 0.69 | 1,02 | 1 | 49 | 1 | 0 | 2 | 0 | 0 | 25 |
| **FI-43** | 57.41 | 2556,58 | 1 | 67 | 1 | 0 | 1 | 0 | 1 | 24 |
| **FI-44** | 543.70 | 26,17 | 1 | 62 | 1 | 0 | 2 | 0 | 1 | 34 |
| **FI-45** | 9.19 | 72,50 | 1 | 58 | 1 | 0 | 1 | 0 | 1 | 33 |
| **FI-46** | 41.16 | 7082,29 | 1 | 70 | 1 | 1 | 1 | 1 | 1 | 24 |
| **FI-47** | 171.65 | 574,04 | 1 | 69 | 1 | 1 | 2 | 1 | 1 | 24 |
| **Sample code** | **KCNH2**  **fold** | **LHR**  **fold** | **FIGO**  **stage^I^** | **Age at diagnosis** | **Histotype^II^** | **Risk^III^** | **Grading ^IV^** | **MI**  **>50%^V^** | **Menopause^VI^** | **BMI** |
| **FI-48** | 52.95 | 600,49 | 1 | 52 | 1 | 0 | 1 | 0 | 0 | 23 |
| **FI-49** | 185.25 | 31,02 | 1 | 48 | 1 | 0 | 1 | 0 | 0 | 24 |
| **FI-50** | 84.64 | 58,28 | 1 | 52 | 1 | 0 | 1 | 0 | 1 | 25 |
| **FI-51** | 61.75 | 266726,20 | 3 | 65 | 1 | 1 | 3 | 1 | 1 | 35 |
| **FI-53** | 370.07 | 4438579,68 | 1 | 68 | 1 |  | 1 | 0 | 1 | 23 |
| **FI-54** | 63.93 | 65,12 | 1 | 73 | 0 | 1 | 3 | 1 | 1 | 33 |
| **FI-55** | 466.26 | 483,82 | 1 | 62 | 1 | 0 | 1 | 0 | 1 | 31 |
| **FI-56** | 359.12 | 729,96 | 1 | 55 | 1 | 0 | 2 | 0 | 1 | 37 |
| **FI-57** | 50.74 | 1,00 | 3 | 72 | 1 | 1 | 2 | 1 | 1 | 27 |
| **FI-58** | 17.67 | 25,63 | 1 | 74 | 1 | 1 | 2 | 1 | 1 | 36 |
| **Fi 59** | 103 | 80,17 | 1 | 78 | 1 | 1 | 2 | 1 | 1 | 37 |
| **FI-60** | 115.09 | 77218,92 | 1 | 50 | 1 | 0 | 2 | 0 | 1 | 27 |
| **FI-61** | 0.09 | 64,15 | 3 | 58 | 1 | 1 | 2 | 1 | 1 | 39 |
| **FI-62** | 110.66 | 15,78 | 1 | 74 | 1 | 1 | 3 | 1 | 1 | 24 |
| **FI-63** | 86.82 | 918,63 | 1 | 67 | 1 | 0 | 1 | 0 | 1 | 23 |
| **FI-65** | 3.96 | 118,88 | 3 | 68 | 1 | 0 | 2 | 0 | 1 | 23 |
| **FI-67** | 20.16 | 181,65 | 1 | 51 | 1 | 0 | 2 | 0 | 1 | 21 |
| **FI-68** | 76.11 | 205,55 | 1 | 49 | 1 | 0 | 2 | 0 | 1 | 26 |
| **FI-69** | 4.56 | 284,71 | 1 | 43 | 1 | 0 | 1 | 0 | 0 | 35 |
| **FI-70** | 531.28 | 1,31 | 1 | 60 | 1 | 1 | 3 | 1 | 1 | 27 |
| **FI-71** | 111.17 | 282,74 | 2 | 45 | 1 | 1 | 3 | 1 | 0 | 21 |
| **FI-72** | 14.16 | 44,53 | 1 | 76 | 1 | 1 | 2 | 1 | 1 | 24 |
| **FI-73** | 10.93 | 84,74 | 1 | 56 | 1 | 0 | 1 | 0 | 1 | 29 |
| **FI-74** | 103.97 | 64,22 | 1 | 76 | 0 | 1 | 3 | 0 | 1 | 25 |
| **FI-75** | 0.23 | 65,80 | 3 | 51 | 0 | 1 | 2 | 1 | 0 | 22 |
| **FI-76** | 141.37 | 17,69 | 1 | 54 | 1 | 0 | 1 | 0 | 1 | 20 |
| **FI-77** | 0.34 | 10,31 | 1 | 68 | 1 | 1 | 3 | 1 | 1 | 39 |
| **FI-78** | 129.79 | 0,82 | 1 | 60 | 1 | 0 | 2 | 0 | 1 | 31 |
| **FI-79** | 9519.48 | 0,76 | 1 | 66 | 1 | 1 | 2 | 1 | 1 | 30 |
| **FI-80** | 2057.49 | 29944,43 | 1 | 39 | 1 | 0 | 1 | 0 | 0 | 33 |
| **FI-81** | 3751.71 | 56789,37 | 1 | 53 | 1 | 1 | 3 | 1 | 1 | 42 |
| **FI-82** | 3821.70 | 2,48 | 1 | 54 | 1 | 1 | 3 | 1 | 1 | 22 |
| **FI-83** | 156.86 | 380,04 | 1 | 75 | 1 | 1 | 1 | 1 | 1 | 24 |
| **FI-84** | 636.20 | 218,78 | 2 | 83 | 1 | 1 | 3 | 1 | 1 |  |
| **FI-85** | 229.13 | 66,56 | 1 | 71 | 1 | 1 | 3 | 1 | 1 | 33 |
| **FI-86** | 10.15 | 2,63 | 1 | 40 | 1 | 0 | 2 | 0 | 0 | 18 |
| **FI-87** | 108.38 | 13,74 | 1 | 80 | 1 | 1 | 3 | 1 | 1 | 22 |
| **FI-88** | 1.33 | 64,59 | 1 | 74 | 1 | 0 | 1 | 0 | 1 | 23 |
| **FI-89** | 16.34 | 38,76 | 1 | 80 | 1 | 1 | 3 | 1 | 1 | 40 |
| **FI-90** | 12.47 | 61,68 | 2 | 80 | 1 | 1 | 3 | 1 | 1 | 31 |
| **FI-92** | 0.94 | 8,92 | 1 | 40 | 1 | 0 | 1 | 0 | 0 | 23 |
| **FI-93** | 1.06 | 0,61 | 1 | 67 | 1 | 1 | 2 | 1 | 1 | 18 |
| **FI-94** | 48.06 | 46,80 | 1 | 48 | 1 | 0 | 2 | 1 | 1 | 23 |
| **Sample code** | **KCNH2**  **fold** | **LHR**  **fold** | **FIGO**  **stage^I^** | **Age at diagnosis** | **Histotype^II^** | **Risk^III^** | **Grading ^IV^** | **MI^V^** | **Menopause^VI^** | **BMI** |
| **FI-95** | 3.32 | 120,54 | 1 | 64 | 1 | 1 | 2 | 1 | 1 | 25 |
| **FI-96** | 348.09 | 4067,71 | 1 | 63 | 1 | 0 | 1 | 0 | 1 | 37 |
| **FI-98** | 34.78 | 30,59 | 1 | 66 | 0 | 1 | 3 | 1 | 1 | 26 |
| **FI-99** | 0.39 | 11,78 | 3 | 61 | 1 | 1 | 3 | 1 | 1 | 23 |
| **FI-100** | 323.29 | 1451,50 | 2 | 71 | 1 | 1 | 2 | 1 | 1 | 38 |
| **FI-102** | 22.52 | 136,87 | 1 | 80 | 1 | 0 | 1 | 0 | 1 | 25 |
| **FI-103** | 29.38 | 3,53 | 1 | 51 | 1 | 0 | 1 | 1 | 1 | 21 |
| **FI-104** | 68.44 | 4,68 | 1 | 68 | 1 | 0 | 2 | 0 | 1 | 25 |
| **FI-105** | 164.28 | 18284,50 | 1 | 39 | 1 | 0 | 1 | 0 | 1 | 26 |
| **FI 107** | 1.71 | 193,56 | 1 | 76 | 1 | 1 | 3 | 1 | 1 | 23 |
| **FI 108** | 1.66 | 51,92 | 1 | 42 | 1 | 1 | 3 | 0 | 0 |  |
| **FI-109** | 13.33 | 701,84 | 1 | 68 | 1 | 0 | 1 | 0 | 1 | 39 |
| **FI 112** | 83.09 | 2886,29 | 3 | 69 | 0 | 1 | 3 | 0 | 1 | 26 |
| **FI 113** | 0.19 | 93,59 | 1 | 83 | 1 | 1 | 2 | 1 | 1 | 23 |
| **FI 115** | 11.82 | 3,25 | 1 | 65 | 0 | 1 | 3 | 0 | 1 | 35 |
| **FI 117** | 5379.79 | 22047,59 | 1 | 78 | 1 | 1 | 2 | 1 | 1 | 37 |
| **FI 118** | 21.96 | 328,18 | 1 | 49 | 1 | 0 | 1 | 0 | 0 | 20 |
| **FI 119** | 5.46 | 728,27 | 1 | 73 | 1 | 1 | 3 | 0 | 1 | 36 |
| **FI 120** | 2385.37 | 75,15 | 1 | 65 | 1 | 0 | 1 | 0 | 1 | 31 |
| **FI 121** | 2830.16 | 7581,85 | 1 | 55 | 1 | 0 | 1 | 0 | 1 | 36 |
| **FI-126** | 22.78 | 5,85 | 1 | 68 | 1 | 1 | 3 | 0 | 1 | 26 |
| **FI-128** | 739.29 | 2112,89 | 1 | 59 | 1 | 0 | 2 | 0 | 1 | 22 |
| **FI-133** | 8014.15 | 53,02 | 1 | 50 | 1 | 0 | 1 | 0 | 1 | 26 |
| **FI-135** | 125.80 | 8,72 | 1 | 59 | 1 | 0 | 1 | 0 | 1 | 34 |
| **Fi 137** | 5969.00 | 32730,17 | 1 | 84 | 0 | 1 | 3 | 1 | 1 | 20 |
| **FI-138** | 7795.00 | 4990,61 | 1 | 59 | 1 | 0 | 1 | 0 | 1 | 25 |
| **FI-139** | 651.07 | 0,67 | 1 | 68 | 1 | 1 | 1 | 1 | 1 | 26 |
| **FI-141** | 153.63 | 8,36 | 1 | 54 | 1 | 0 | 2 | 0 | 1 |  |
| **FI-142** | 0.44 | 934,68 | 1 | 65 | 1 | 0 | 2 | 0 | 1 | 26 |
| **FI-143** | 165.04 | 36,59 | 1 | 74 | 1 | 1 | 2 | 1 | 1 | 29 |
| **FI-145** | 13.39 | 9,68 | 1 | 76 | 1 | 1 | 2 | 1 | 1 | 24 |
| **FI-146** | 0.40 | 405,91 | 1 | 62 | 1 | 1 | 3 | 1 | 1 | 27 |
| **FI-149** | 98.47 | 0,79 | 1 | 62 | 1 | 1 | 2 | 1 | 1 | 42 |
| **FI-150** | 18.70 | 85,53 | 1 | 56 | 1 | 0 | 2 | 0 | 1 | 32 |
| **FI-152** | 70077.64 | 186007,34 | 1 | 72 | 1 | 0 | 2 | 0 | 1 | 31 |
| **FI-153** | 309.40 | 10202,72 | 1 | 78 | 1 | 0 | 2 | 0 | 1 | 23 |
| **Fi-154** | 70 | 502930,00 | 2 | 80 | 1 | 1 | 3 | 1 | 1 |  |
| **FI-155** | 174.05 | 12431,10 | 1 | 66 | 1 | 0 | 1 | 0 | 1 | 51 |
| **FI-156** | 663.22 | 142,68 | 1 | 75 | 1 | 0 | 2 | 0 | 1 | 30 |
| **FI-157** | 22484.87 | 2882,96 | 1 | 76 | 0 | 1 | 3 | 0 | 1 | 28 |
| **FI-158** | 2110.45 | 53,32 | 1 | 63 | 1 | 0 | 2 | 0 | 1 | 25 |

**Supplementary Table S10:** The expression of LH-R and KCNH2 mRNA is reported for 126 samples deriving from patient affected with EC. In the table are reported also the clinic-pathological parameters of each patient (**^I^**: Figo stage IA and IB=1; Figo stage II=2; Figo stage IIIA, IIIB, IIIC=3; Figo stage IV=4. **^II^**: Endometrioid histotype=1, Non Endometrioid histotype=0. **^III^**: Low risk=0, High risk=1. **^IV^**: Grading G1=1: Grading G2=2; Grading G3=3. **^V^**: MI (myometrial invasion)>50%=1; MI <50%=0. **^VI^**: Menopause at the diagnosis no=0; Menopause at the diagnosis yes=1).

| **Supplementary Table S11** | | | | |
| --- | --- | --- | --- | --- |
| **Sample name** | **Genotype information** | **Specimen type** | **Age of mice** | **Sample description** |
| US45103023_252665519150_S02_GE1-v1_95_Feb07_1_3 | TG-LHR-frt-200 | Uterus | 6 months | Healthy tissue |
| US45103023_252665519150_S02_GE1-v1_95_Feb07_1_4 | TG-LHR-frt-200 | Uterus | 6 months | Healthy tissue |
| US45103023_252665519150_S02_GE1-v1_95_Feb07_1_1 | WT | Uterus | 6 months | Healthy tissue |
| US45103023_252665519150_S02_GE1-v1_95_Feb07_1_2 | WT | Uterus | 6 months | Healthy tissue |
| US45103023_252665519151_S01_GE1-v5_95_Feb07_1_1 | TG-LHR-frt-200 | Uterine tumor mass | 17 months | Carcinoma |
| US45103023_252665519151_S01_GE1-v5_95_Feb07_1_2 | WT | Uterus | 17 months | Healthy tissue |
| US45103023_252665519151_S01_GE1-v5_95_Feb07_1_3 | WT | Uterus | 17 months | Healthy tissue |
| US45103023_252665519151_S01_GE1-v5_95_Feb07_1_4 | TG-LHR-frt-200 | Uterus | 17 months | Healthy tissue |

**Supplementary Table S11:** Sample name, genotypes information, specimen types and ages of mice used for gene expression analysis are resumed in the present table. These information paper have been deposited in Gene Expression Omnibus (GEO) and are accessible through the accession number GSE163488.
